# Continuous Bioinspired Oxidation of Sulfides

**DOI:** 10.3390/molecules25112711

**Published:** 2020-06-11

**Authors:** Francesca Mangiavacchi, Letizia Crociani, Luca Sancineto, Francesca Marini, Claudio Santi

**Affiliations:** Department of Pharmaceutical Sciences (Group of Catalysis, Synthesis and Organic Green Chemistry), University of Perugia via del Liceo, 1–06123 Perugia, Italy; francesca.mangiavacchi@studenti.unipg.it (F.M.); letizia.crociani@studenti.unipg.it (L.C.); luca.sancineto@unipg.it (L.S.); francesca.marini@unipg.it (F.M.)

**Keywords:** selenium dioxide, sulfide, sulfoxide, sulfone, flow chemistry, catalysis, oxidation, hydrogen peroxide

## Abstract

A simple, efficient, and selective oxidation under flow conditions of sulfides into their corresponding sulfoxides and sulfones is reported herein, using as a catalyst perselenic acid generated in situ by the oxidation of selenium (IV) oxide in a diluted aqueous solution of hydrogen peroxide as the final oxidant. The scope of the proposed methodology was investigated using aryl alkyl sulfides, aryl vinyl sulfides, and dialkyl sulfides as substrates, evidencing, in general, a good applicability. The scaled-up synthesis of (methylsulfonyl)benzene was also demonstrated, leading to its gram-scale preparation.

## 1. Introduction

Heteroatom oxidation, with particular emphasis on nitrogen and sulfur, is a process of great relevance in organic synthesis. For the oxidation of sulfides into their corresponding sulfoxides and sulfones, a number of procedures have been described, including stoichiometric reactions, chemocatalytic reactions, and bio-catalytic reactions [1]. The main goal of this kind of transformation is the selective preparation of the mono-oxygenated over the bis-oxygenated derivatives, and the result is strongly dependent on the protocol used for the oxidation, being directly correlated to the nucleophilicity of the sulfide and sulfoxide and the electrophilicity of the actual oxidizing species.

Arylsulfones and arylsulfoxides are useful synthetic intermediates and building blocks for the synthesis of a series of biologically active compounds [2,3].

**Figure 1:**
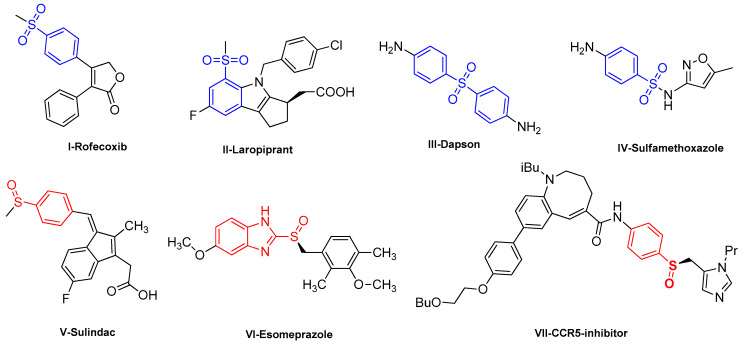
Selected examples of aryl sulfones and aryl sulfoxides.

A non-exhaustive panel of examples is presented in Figure 1: an arylsulfone moiety is present in drugs like Rofecoxib (**I**) [4], Laropiprant (**II**) [5], Dapson (**III**) [6], and Sulfamethoxazole (**IV**) [7], while examples of arylsulfoxides (even if less distributed due to their relative instability) can be found in Sulindac (**V**) a clinically used anti-inflammatory drug [8], in esomeprazole (**VI**), the *S* enantiomer of omeprazole [9], and in a recently reported compound (**VII**) with anti-HIV activity [10].

Over the last ten years, we have studied the applicability of selenium derivatives as catalysts in biomimetic oxidations, demonstrating that this approach presents a number of advantages in terms of eco-sustainability [11].

We demonstrated that as the selenium atom of the glutathione peroxidase enzyme catalyzes the oxidation of two molecules of glutathione using peroxides as stoichiometric oxidants, small organoselenium molecules can be biomimetically used as catalysts in the hydrogen-peroxide-mediated oxidation of thiols [12]. The same reactivity can be translated into the oxidation of carbon–carbon double bonds, affording epoxide intermediates that can be opened by a nucleophilic solvent, affording vicinal diols [13,14] or α-methoxy alcohols [14] when the reaction is carried out in water or in methanol, respectively. When the substrate is an alkenol or an alkenoic acid, the epoxydic intermediate is opened intramolecularly by the internal nucleophile, leading to oxidative cyclofunctionalization with the formation of cyclic hydroxylethers or hydroxylactones [15]. Furthermore, the same catalytic system has been used also to oxidize aldehydes into their corresponding carboxylic acids in “on water” conditions, or into their corresponding esters when the reaction medium is a primary or a secondary alcohol [16].

The possibility of performing these catalytic reactions in aqueous media or in biphasic systems and the beneficial effects on the reactivity and selectivity, as well as the reaction workup, product purification, and recovery and reuse of the catalyst, were recently reviewed [17].

Nowadays, continuous-flow transformations represent an innovative methodology which is gaining increasing interest in both academic and industrial research including in the pharmaceutical industry. Reactions performed under continuous flow are generally faster, safer, and more environmentally friendly than those realized in batches. Recently, great progress was also made in terms of flexibility and robustness for large-scale processes [18], as well as in bioorganic catalysis [19], photochemistry [20], and electrochemistry, combined with on-line analysis [21,22]. For these reasons, studies focused on the reinterpretation of old transformations with this new technology are of particular interest in order to increase the number of synthetic tools exploitable in flow chemistry.

After a seminal study in which we demonstrated that bioinspired selenium-catalyzed reactions can be translated into continuous mode [23] some of the present authors, using a biphasic liquid–liquid system, reported the continuous oxidative cyclofunctionalization of alkenoic acids. The reaction conditions were optimized for the synthesis and purification of a library of biologically relevant lactones using a fully automated flow setup [24]. We clearly observed that the flow conditions produced a sensible reduction of the reaction time (residence time) as a consequence of a slug flow, which created local vortex fields able to increase the mass transfer rates between the organic and the aqueous phases. Here, we report our recent results in the selenium-catalyzed oxidation of sulfides under flow conditions to selectively afford sulfoxides and sulfones in line with the principles of efficiency, safety, mild reaction conditions, and overall atom economy.

The first example of a continuous oxidation of sulfides was reported by Nunez in 2010, who used silica-supported peracid and supercritical carbon dioxide and obtained a moderate level of chemoselectivity depending on the pressure [25]. In the same period, a green method was reported for the continuous and chemoselective synthesis of a few arylsulfoxides using diluted hydrogen peroxide and the sulfonic resin Amberlite 120 H [26].

More recently, a selective method based on the use of a microflow electrocell for electrochemical oxidation was reported, in which the selectivity can be tuned by changing the applied potential [27], and a method in which a solution of sulfide in dichloromethane and 15 vol. % of CF_3_COOH were fluxed in a packed-bed reactor filled with Oxone^®®^. In the latter case, an example of selective mono-oxygenation of a disulfide was also described [28].

**Scheme 1:**
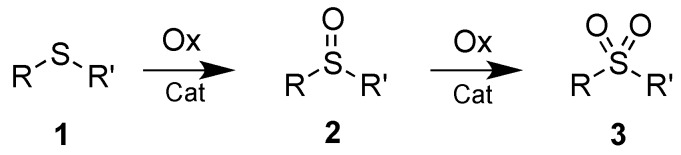
The oxidation of sulfide **1** into the corresponding sulfoxide **2** and sulfone **3**.

In our protocol, a bioinspired approach was implemented for the continuous and chemoselective oxidation of sulfides (**1**) into their corresponding sulfoxides (**2**) or sulfones (**3**) (Scheme 1). The reaction was carried out using SeO_2_ as the most atom-economical Se-based pre-catalyst, in a liquid–liquid biphasic system at room temperature with hydrogen peroxide as the green oxidant.

## 2. Results and Discussions

Some years ago, Drabowicz et al. reported that the synthesis of sulfoxides [29] and sulfones [30] can be achieved by starting from the corresponding sulfides, using hydrogen peroxide/selenoxide system to form peroxyselenic (IV) acid in situ as the actual stoichiometric oxidizing reagent. Even if the synthetic versatility of selenium (IV) oxide is widely reported in the literature, its use in organic synthesis, especially as a stoichiometric reagent, is often limited by its toxicity and tendency to generate volatile and malodorous side products [31]. With the aim of overcoming these limitations, we decided to explore the possibility of using SeO_2_ as the catalyst in a bioinspired oxidation process in the presence of hydrogen peroxide using liquid–liquid biphasic flow conditions. Preliminary investigations were carried out by following the conversion of phenylethylsulfide **1a** into its sulfoxide (**2a**) and sulfone (**3a**). The effects of the flow rate and the amount of hydrogen peroxide were evaluated, looking at the conversion as well as the chemoselectivity of the reaction.

**Scheme 2:**
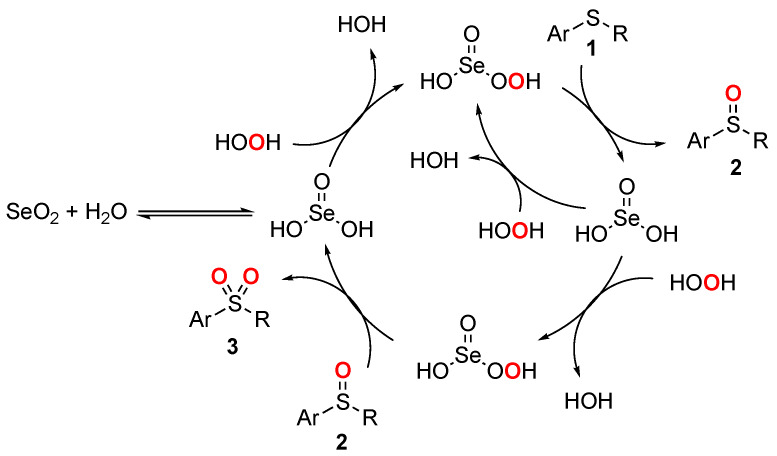
Proposed catalytic mechanism.

A 0.5 M solution of the substrate **1a** in ethyl acetate (Solution A) and a 0.05 M solution of SeO_2_ in water in the presence of the different amounts of H_2_O_2_ reported in Table 1 (Solution B), were fluxed through a Y junction in a 2 mL tubular reactor, at four different flow rates corresponding to residence times of 6.5, 10, 20 and 100 min. The chemoselectivity seemed to be influenced predominantly by the amount of hydrogen peroxide (Entries 1–5 vs. Entries 6–8). In entries 3–5 the conversion yield for the formation of the sulfoxide **2a** increased with the reduction of the flow rate from 0.3 mL/min to 0.1 mL/min and, in these conditions, using 2 equivalents of hydrogen peroxide, the target compound (**2a**) was obtained in an 85% yield and with 100% chemoselectivity. When the reaction was repeated in the same conditions but without SeO_2_, only the starting material was observed in the ^1^H-NMR spectrum of the crude product, confirming the catalytic role of selenium. Increasing the amount of hydrogen peroxide from 2 (entry 5) to 5 (entry 7) and 10 (entries 7,8) equivalents also increased the formation of the sulfone **3a**, which was quantitatively obtained as a unique reaction product when the reaction was carried out in the presence of 10 equivalents of H_2_O_2_ at both 0.1 and 0.2 mL/min (entries 7,8). The proposed mechanism for the catalytic cycle is reported in Scheme 2. Selenium (IV) oxide in water is in equilibrium with the hydrated form which is particularly prone to oxidation by hydrogen peroxide, affording the corresponding peroxyselenic (IV) acid as the actual catalyst in the formation of both the sulfoxide **2** and sulfone **3**, depending on the amount of the oxidant.

From these preliminary data, the reaction conditions reported in Entry 5 and Entry 8 were selected as the best ones for the evaluation of the scope. A set of sulfides (Compounds **1a**–**g**, Table 2) was converted into the corresponding sulfoxides **2a**–**f** and sulfones **3a**–**g**.

The results obtained for the synthesis of sulfoxides **2a**–**g** are summarized in Table 2. All the tested aryl sulfides **1a**–**f** afforded the corresponding mono-oxygenated derivatives **2a**–**f** in very high yields and with complete chemoselectivity. Only in the case of the naphtylethylsulfide **1d** was it necessary to use diluted solutions, due to a lower solubility leading to poor conversion into the target compound **2d** (20%). In order to increase the yield, we decided to use 5 equivalents of hydrogen peroxide. In these new conditions, **1d** was quantitatively converted in an 80:20 mixture of **2d/3d**, from which **2d** was isolated in a 75% yield after chromatographic purification by flash chromatography. In the case of the aliphatic tetrahydrothiofene **1g**, it was impossible to optimize the conditions for the synthesis and purification of sulfoxide **2g**, and a mixture of **1g**, **2g**, and **3g** was obtained that proved to be neither separable by chromatography nor unequivocally quantifiable by ^1^H NMR.

Excellent results were also obtained for the synthesis of the sulfones **3a**–**g**, (Table 3). In all the cases, the desired products were obtained pure and in quantitative yield simply by the separation of the organic layers and the removal under vacuum of the solvent, without the need for any other purification. In the cases of substrate **1d**, we used a 0.25 M solution of the starting material and a 0.025 M solution of the catalyst to overcome the solubility issues of the corresponding sulfone **3d**, without any detrimental effect on the yield.

In these conditions, the aliphatic substrate **1g** was quantitatively converted into the corresponding sulfone **3g**, which was isolated and characterized using ^1^H and ^13^C-NMR spectroscopy.

It was observed that in the case of vinyl sulfide **1e**, the proposed methodology afforded only the oxidation of the sulfur atom, and in both conditions for the synthesis of the sulfoxide **2d** or the sulfone **3d**, the C=C was not oxidized.

Finally, in order to test the scalability of the process, 10 mmol of sulfide **1b** was processed using the conditions reported in Table 3 affording, in 200 min, quantitatively a 91:9 mixture of 3**b**/2**b** corresponding to a processing rate of 10.16 gd^−1^ for **3b**. (See Supplementary Materials).

## 3. Materials and Methods

Solvents and reagents were purchased from Sigma-Aldrich (St Louis, MS, USA), Alfa Aesar (Kandel, Germany), and VWR (Milano, Italy), and used as received unless otherwise noted. Compound **1d** was prepared according to the procedure reported in literature. [32] Analytical thin-layer chromatography (TLC) was performed on silica-gel-precoated (60F-254) aluminum foil sheets (Merck, Darmstadt, Germany), using short-wave UV light (TUV T8 HO 95W G13 UVC, Philips, Milano, Italy) as the visualizing agent. NMR spectroscopic (Bruker, Fällanden, Switzerland) experiments were conducted at 25 °C on Bruker DPX and DRX spectrometers operating at the specified frequencies. ^1^H and ^13^C chemical shifts (δ) are reported in parts per million (ppm), relative to TMS (*δ* = 0.0 ppm) and the residual solvent peak of CDCl_3_ (*δ* = 7.27 and 77.00 ppm in ^1^H and ^13^C NMR, respectively). Data are reported as chemical shift (multiplicity, coupling constants where applicable, number of hydrogen atoms). Abbreviations are: s (singlet), d (doublet), t (triplet), q (quartet), dd (doublet of doublet), dt (double of triplet), tt (triplet of triplet), m (multiplet), br. s (broad signal). Coupling constants (*J*) are quoted in Hertz (Hz) to the nearest 0.1 Hz. Melting points were measured using a Kofler hot-stage-microscope Thermovar (Reichert, Vienna, Austria) and are reported as uncorrected data.

### 3.1. Synthesis of Sulfoxides 2a–f

In a 2 mL volumetric flask, ethyl acetate was added to sulfides 1a–c,e–g (1.0 mmoL) or sulfide 1d (0.5 mmoL) to make up the required volume. (Solution A: 0.5 M (1a–c,e–g). In the case of 1d the concentration was optimized at 0.25 M.) Into a 2 mL volumetric flask, 0.1 mmol of SeO_2_ (11 mg) and H_2_O_2_ 30 wt. % (2 mmol, 205 µL) were poured, and then water was added until the desired volume was reached. (Solution B: 0.05 M (SeO_2_) and 1 M (H_2_O_2_). In the case of 1d, a different concentration was optimized at 0.025 M (SeO_2_) and 1.25 M (H_2_O_2_)).

Solutions A and B were fluxed at the same flow rate through a multi-syringe pump apparatus (Chemyx Fusion 100, Stafford, TX, USA) equipped with two syringes (2 mL), and they were mixed in a Y-junction and flowed at 0.100 mL/min through a tubularPTFE reactor coil with internal diameter of 1 mm and internal volume of 2 mL (Bohlender GmbH, Grünsfeld, Germany) at room temperature. The system was washed with 4 mL of ethyl acetate, and the aqueous and organic layers were collected in a flask, quenched with an aqueous solution of Na_2_S_2_O_3_ 10% (*w*/*v*) and extracted with ethyl acetate (5 mL × 3). The combined organic layers were dried over sodium sulfate and concentrated under reduce pressure, affording the target sulfoxides. When required, the unreacted starting material was removed under vacuum, and in the case of 2d, it was separated from the corresponding sulfone (3d) by flash chromatography on a silica gel column using EtOAc/petroleum ether (2:98) as eluent.

### 3.2. Synthesis of Sulfones 2a–g

In a 2 mL volumetric flask, ethyl acetate was added to sulfides 1a–c,e–g (1.0 mmoL) or sulfide 1d, (0.5 mmoL) to make up the required volume. (Solution A: 0.5 M (1a–c,e–g). In the case of 1d, the concentration was optimized at 0.25 M) In a 2 mL volumetric flask, 0.1 mmol of SeO_2_ (11 mg) and H_2_O_2_ 30 wt. % (10 mmoL, 1.025 mL) were poured, and then water was added until the desired volume was reached. (Solution B: 0.05 M (SeO_2_) and 5 M (H_2_O_2_). In the case of 1d, a different concentration was optimized at 0.025 M (SeO_2_) and 2.5 M (H_2_O_2_)).

Solutions A and B were fluxed at the same flow rate through a multi-syringe pump apparatus equipped with two syringes (2 mL), and they were mixed in a Y-junction and flowed at 0.200 mL/min through a tubular PTFE reactor coil with internal diameter of 1 mm and internal volume of 2 mLat room temperature. The system was washed with 4mL of ethyl acetate, and the aqueous and organic layers were collected in a flask, quenched with an aqueous solution of Na_2_S_2_O_3_ 10% (*w*/*v*), and extracted with ethyl acetate (5 mL × 3). The combined organic layers were dried over sodium sulfate and concentrated under reduce pressure affording the target sulfones without further purification.

### 3.3. Spectral Data

Figures of NMR spectra of all the synthesized compounds are reported in the Supplementary Materials

 

Ethyl(phenyl)sulfoxide (2a) [33]: pale yellow oil (131 mg, 0.85 mmoL, yield 85%).^1^H-NMR (400 MHz, CDCl_3_): δ 7.64–7.53 (m, 5H), 2.96–2.89 (m, 1H), 2.82–2.77 (m, 1H), 1.22 (t, *J* = 7.38 Hz, 3H) ppm. ^13^C-NMR (50 MHz, CDCl_3_): δ 143.2, 131.0, 50.3, 124.2, 129.2, 6.0, ppm.

 

Methyl(phenyl)sulfoxide (2b) [33]: pale red oil (127 mg, 0.9 mmoL, yield 91%).^1^H-NMR (400 MHz, CDCl_3_): δ 7.58–7.56 (m, 2H), 7.46–7.44 (m, 3H), 2.65 (s, 3H) ppm. ^13^C-NMR (50 MHz, CDCl_3_): δ 145.6, 131.1, 129.4, 123.5, 44.0 ppm.

 

Methyl(4-methylphenyl)-sulfoxide (2c) [33]: pale yellow oil (137.1 mg, 0.9 mmoL, yield 89%).^1^H-NMR (200 MHz, CDCl_3_): δ 7.58–7.54 (m, 2H), 7.37–7.32 (m, 2H), 2.72 (s, 3H), 2.44 (s, 3H) ppm. ^13^C-NMR (50 MHz, CDCl_3_): δ 142.4, 141.5, 130.0, 123.5, 44.0, 21.4 ppm.

 

Ethyl(naphthalen-2-yl)sulfoxide (2d) [32]: pale yellow oil (82 mg, 0.375 mmoL, yield 75%).^1^H-NMR (200 MHz, CDCl_3_): δ 8.16 (s, 1H), 8.96–7.87 (m, 3H), 7.57-7.53 (m, 3H), 3.02–2.93 (m, 1H), 2.86–2.77 (m, 1H), 1.19 (t, *J = 7.4* Hz, 3H) ppm. ^13^C-NMR (50 MHz, CDCl_3_): δ 140.3, 134.4, 132.8, 129.4, 128.5, 128.1, 127.7, 127.3, 125.0, 120.0, 6.0, 49.9 ppm.

 

Phenyl(ethenyl)sulfoxide (2e) [34]: pale yellow oil (112 mg, 0.7 mmoL, yield 74%).^1^H-NMR (400 MHz, CDCl_3_): δ 7.56 -7.54 (m, 2H), 7.45-7.43 (m, 3H), 6.53 (dd, *J* = 9.5 Hz and J = 16.5 Hz, 1H), 6.14 (d, *J* = 16.4 Hz, 1H), 5.83 (d, *J* = 9.6 Hz, 1H) ppm. ^13^C-NMR (50 MHz, CDCl_3_): δ 143.3, 142.9, 131.3, 129.5, 124.7, 120.8 ppm.

 

Phenyl(benzyl)sulfoxide(2f) [35]: white crystals (m.p. 120–122 °C, lit 124–126 °C) [36]: (216 mg, 1 mmoL, quantitative yield).^1^H-NMR (200 MHz, CDCl_3_): δ 7.44–7.24 (m, 8H), 6.99–6.96 (m, 2H), 4.12–3.95 (m, 2H) ppm. ^13^C-NMR (50 MHz, CDCl_3_): δ 142.4 13.5, 130.6, 128.9, 128.5, 128.4, 128.2, 124.2, 63.2, ppm.

 

(Ethylsulfonyl)benzene (3a) [37]: white crystals (m.p. 39–42 °C, lit 39–41 °C) [37] (168 mg, 1 mmol, yield > 99%).^1^H-NMR (400 MHz, CDCl_3_): δ 7.87–7.82 (m, 2H), 7.61-7.47 (m, 3H), 3.06 (q, *J* = 6.9 Hz, 2H), 1.21 (t, *J* = 6.9 Hz, 3H) ppm. ^13^C-NMR (50 MHz, CDCl_3_): δ 138.4, 133.8, 129.3, 128.2, 50.6, 7.5, ppm.

 

(Methylsulfonyl)benzene (3b) [37]: white solid (m.p. 84–86 °C, lit 85–87 °C) [37] (156 mg, 1 mmoL, yield > 99%). ^1^H-NMR (200 MHz, CDCl3): δ 7.99-7.96 (m, 2H), 7.73-7.56 (m, 3H), 3.08 (s, 3H) ppm. ^13^C-NMR (50 MHz, CDCl_3_): δ 140.5, 133.7, 129.4, 127.4, 44.5 ppm.

 

1-Methyl-4-(methylsulfonyl)benzene (3c) [37]: white crystals (m.p. 85–87 °C, lit 82–85 °C) [37] (169 mg, 1 mmol, yield >99%).^1^H-NMR (200 MHz, CDCl_3_): δ 7.87-7.83 (m, 2H), 7.41-7.36 (m, 2H), 3.05 (s, 3H), 2.46 (s, 3H) ppm. ^13^C-NMR (50 MHz, CDCl_3_): δ 144.7, 137.7, 130.0, 127.4, 44.6, 21.7 ppm.

 

2-(Ethylsulfonyl)naphthalene (3d) [32]: brown oil (109.8 mg, 0.5 mmoL, yield > 99%).^1^H-NMR (200 MHz, CDCl_3_): δ 8.36 (s, 1H), 7.89-7.72 (m, 4H), 7.54-7.47 (m, 2H), 3.07 (q, *J* = 7.0 Hz, 2H), 1.16 (t, *J* = 7.0 Hz, 3H) ppm. ^13^C-NMR (50 MHz, CDCl_3_): δ 7.3, 50.3, 122.5, 127.5, 127.8, 128.1, 128.3, 128.7, 129.1, 129.1, 129.4, 129.8, 130.6, 131.9, 135.0.

 

(Vinylsulfonyl)benzene (3e) [34]: white crystals m.p. (64–65 °C lit 64–65 °C) [34]: (167 mg, 1 mmol, yield > 99%).^1^H-NMR (200 MHz, CDCl_3_): δ 7.84–7.82 (m, 2H). 7.58–7.47 (m, 3H), 6.60 (dd, *J* = 9.8 Hz, 16.5 Hz, 1H), 6.40 (d, *J* = 16.5 Hz, 1H), 5.98 (d, *J* = 9.8 Hz, 1H) ppm. ^13^C-NMR (50 MHz, CDCl_3_): δ 139.5 138.4, 133.7, 129.4, 127.9, 127.8 ppm.

 

(Benzylsulfonyl)benzene (3f) [35]: white crystals (m.p. 147–149 °C, lit 147–149 °C) [38]: (231 mg, 1 mmol, yield > 99%).^1^H-NMR (200 MHz, CDCl_3_): δ 7.57–7.52 (m, 3H), 7.40–7.37 (m, 2H), 7.26–7.19 (m, 3H), 7.02-7.01 (m, 2H), 4.25 (s, 2H) ppm. ^13^C-NMR (50 MHz, CDCl_3_): δ 137.8, 133.8, 130.8, 128.9, 128.8, 128.7, 128.6, 128.1, 62.9 ppm.

 

Tetrahydrothiophene 1,1-dioxide (3g) [37]: pale yellow oil (120 mg, 1 mmoL, yield > 99%).^1^H-NMR (200 MHz, CDCl_3_):δ 3.08–3.01 (m, 2H), 2.27-2.20 (m, 2H).^13^C-NMR (50 MHz, CDCl_3_): δ 51.2, 22.8, ppm.

## 4. Conclusions

We demonstrated herein that, following a bioinspired approach, SeO_2_ can be conveniently used as pre-catalyst in the chemoselective preparation of sulfoxides and sulfones under flow conditions. The use of hydrogen peroxide as final oxidant allowed the safe in situ generation of perselenic acid as the actual catalyst, affording the target compounds in good to excellent yields. The proposed protocol is particularly attractive in terms of its simplicity, and further demonstrates that organoselenium catalysts can be conveniently employed in continuous oxidation reactions using liquid–liquid biphasic systems, improving the safety, efficiency, and the greenness of these processes.

## Figures and Tables

**Table 1:**
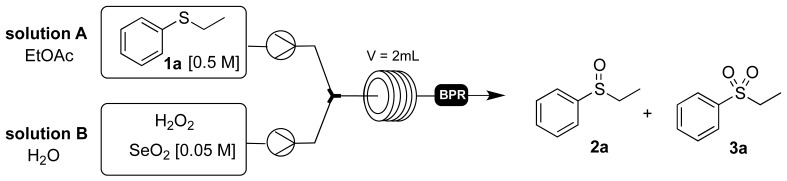
Results of the preliminary screening of the flow-rate conditions obtained by fluxing Solution A (**1a** [0.5 M] in EtOAc) and Solution B (SeO_2_ [0.05 M] and H_2_O_2_ in H_2_O) at room temperature, each at the half-total flow rate.

Entry	H_2_O_2_ eq [conc]	Total Flow RatemL/min	Residence Time (min)	NMR Conversion %	Selectivity 2/3
1	1 [0.5 M]	0.3	6.5	59%	95:5
2	1 [0.5 M]	0.02	100	61%	100:0
3	2 [1.0 M]	0.3	6.5	60%	100:0
4	2 [1.0 M]	0.2	10	78%	100:0
5	2 [1.0 M]	0.1	20	85%	100:0
6	5 [2.5 M]	0.1	20	>99%	71:29
7	10 [5.0 M]	0.1	20	>99%	0:100
8	10 [5.0 M]	0.2	10	>99%	0:100

**Table 2:**
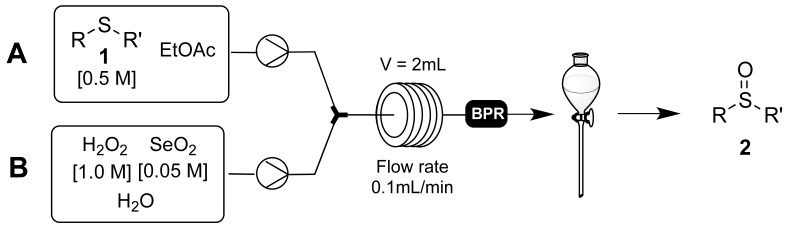
Flow synthesis of sulfoxides **2a**–**f**. (Each solution was fluxed at 0.05 mL/min.).

Substrate	Product	NMR Conversion (Isolated Yield) 2 ^1^	Selectivity 2/3 ^1^
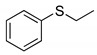	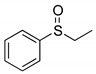	85% (85%)	100:0
**1a**	**2a**		
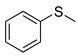	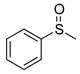	91% (91%)	100:0
**1b**	**2b**		
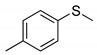	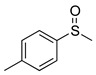	93% (89%)	100:0
**1c**	**2c**		
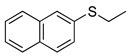	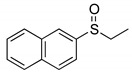	20% 99% ^2^(75%)	100:0 80:20
**1d**	**2d**		
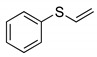	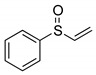	80% (74%)	100:0
**1e**	**2e**		
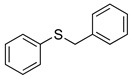	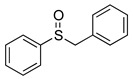	99% (99%)	100:0
**1f**	**2f**		
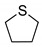	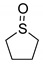	n.d. ^3^	n.d. ^3^
**1g**	**2g**		

^1^ Conversion from the crude product, evaluated by ^1^H-NMR, into the isolated yields shown in brackets;; ^2^ Solution A (**1d,** [0.25 M] in EtOAc), Solution B (SeO_2_ [0.025 M] and H_2_O_2_ [1.25 M] in H_2_O). ^3^ Not determined (a non-resolvable mixture of **1g**, **2g**, and **3g** was obtained, and it was not possible to calculate the corresponding ratio from the ^1^H-NMR spectrum of the crude product).

**Table 3:**
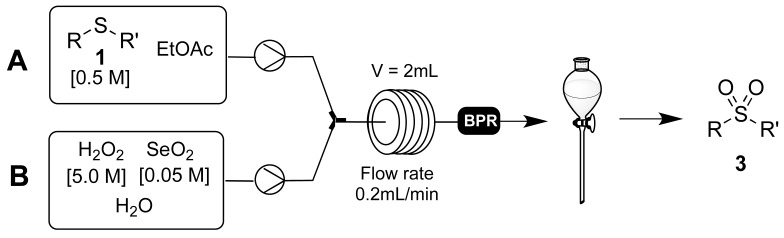
Flow synthesis of sulfones **3a**–**g**. (Each solution was fluxed at 0.1 mL/min).

Substrate	Product	Yield of 3
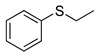	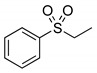	> 99%
**1a**	**3a**	
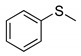	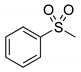	> 99%
**1b**	**3b**	
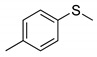	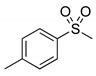	> 99%
**1c**	**3c**	
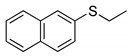	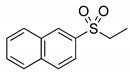	> 99% ^1^
**1d**	**3d**	
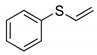	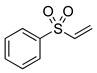	> 99%
**1e**	**3e**	
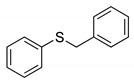	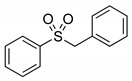	> 99%
**1f**	**3f**	
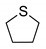	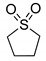	> 99%
**1g**	**3g**	

^1^ To avoid precipitation of the reaction product, diluted solutions were used: Solution A (**1d** [0.25 M] in EtOAc), Solution B (SeO_2_ [0.025 M] and H_2_O_2_ [2.5 M] in H_2_O).

## Supplementary Materials

The following are available online at https://www.mdpi.com/1420-3049/25/11/2711/s1. Figures of NMR spectra of all the synthesized compounds.
