# Supplementary material for: Continuous Bioinspired Oxidation of Sulfides
Source: Molecules. 2020 Jun 11;25(11):2711. doi: 10.3390/molecules25112711 (PMC7321102; doi:10.3390/molecules25112711)

**SUPPORTING INFORMATIONS**

# Continuous Bioinspired Oxidation of Sulfides

**Francesca Mangiavacchi, Letizia Crociani, Luca Sancineto, Francesca Marini and Claudio Santi ***

Department of Pharmaceutical Sciences (Group of Catalysis, Synthesis and Organic Green Chemistry) University of Perugia via del Liceo, 1 – 06123 Perugia – Italy

NMR spectra of compounds **2a-f** and **3a-g** S-2
NMR of the crude from the scale up oxidation of **1b** S-15

**2a**

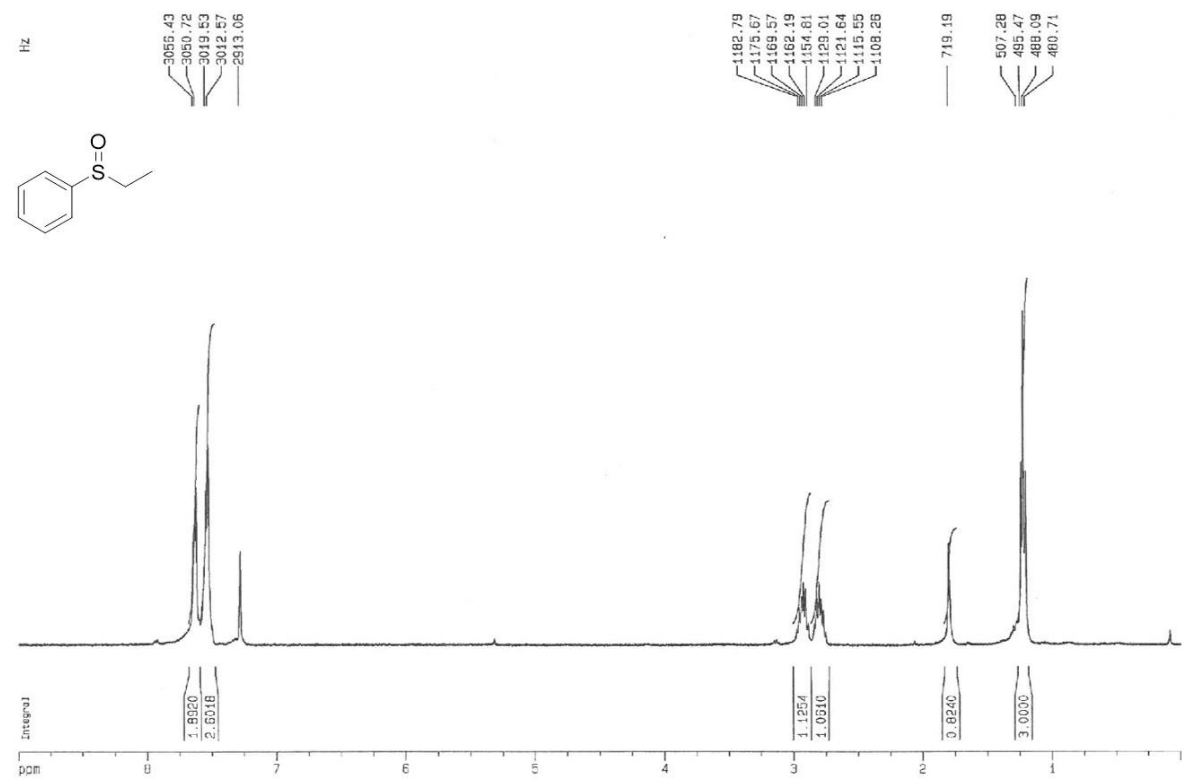
Hz
3056.43
3050.72
3019.53
3012.57
2913.06
1182.79
1175.67
1169.57
1162.19
1154.81
1129.01
1121.64
1115.55
1108.26
719.19
507.28
495.47
488.09
480.71
O
S
Integral
1.8920
2.6018
1.1254
1.0610
0.8240
3.0000
ppm
8
7
6
5
4
3
2
1

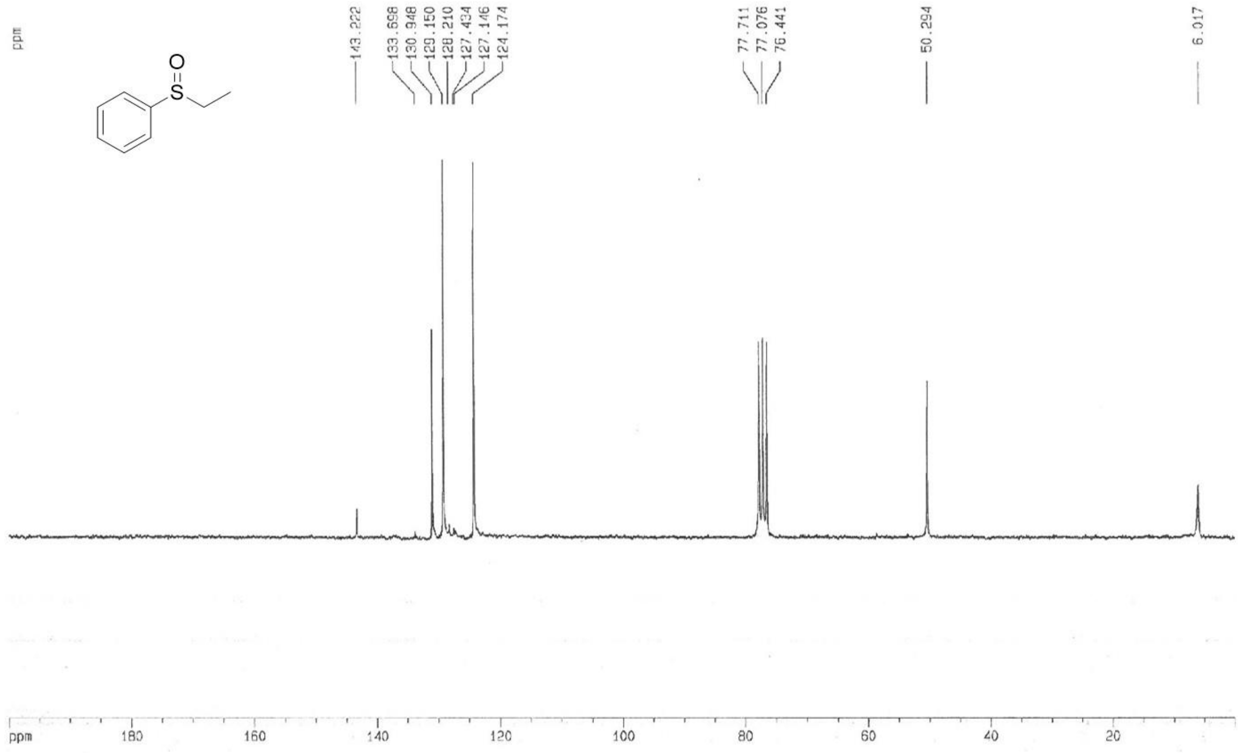
ppm
143.222
133.698
130.948
129.150
128.210
127.434
127.146
124.174
77.711
77.076
76.441
50.294
6.017
O
S
ppm
180
160
140
120
100
80
60
40
20

**3a**

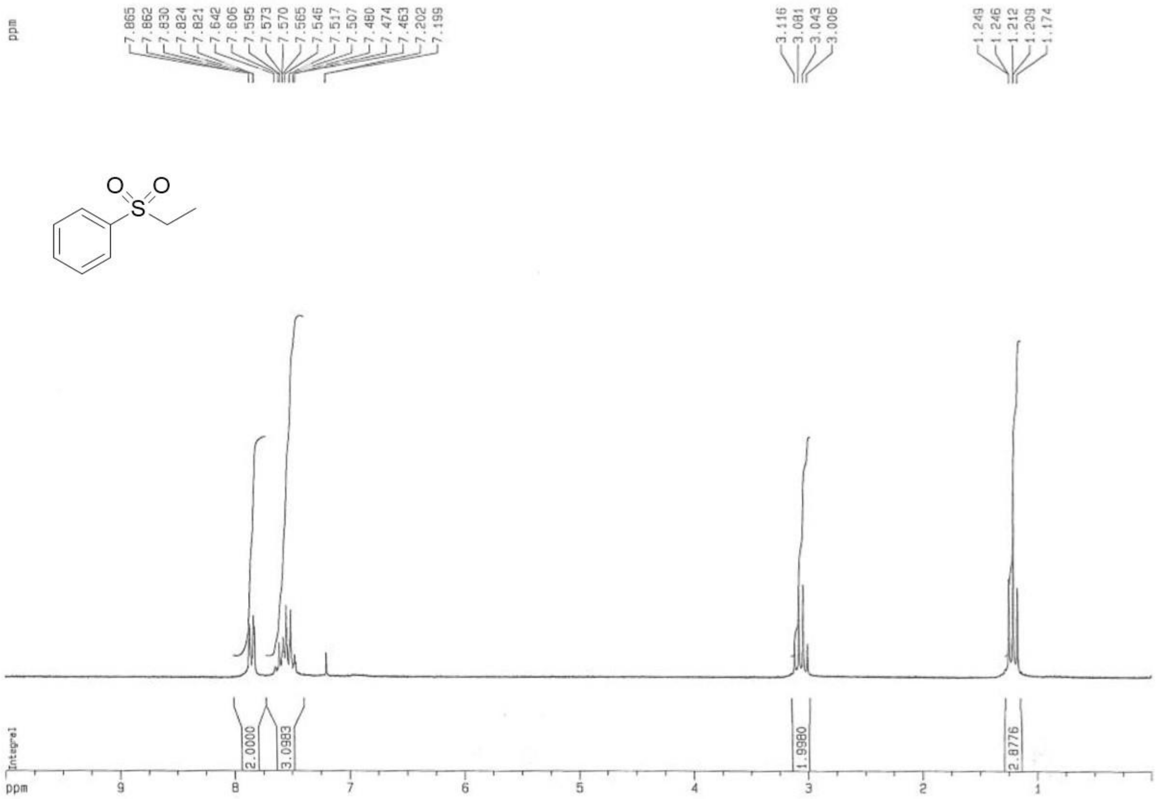
ppm
7.865
7.862
7.830
7.824
7.821
7.642
7.606
7.595
7.573
7.570
7.565
7.546
7.517
7.507
7.480
7.474
7.463
7.202
7.199
3.116
3.081
3.043
3.006
1.249
1.246
1.212
1.209
1.174
Integral
2.0000
3.0983
1.9980
2.8776
ppm
9
8
7
6
5
4
3
2
1

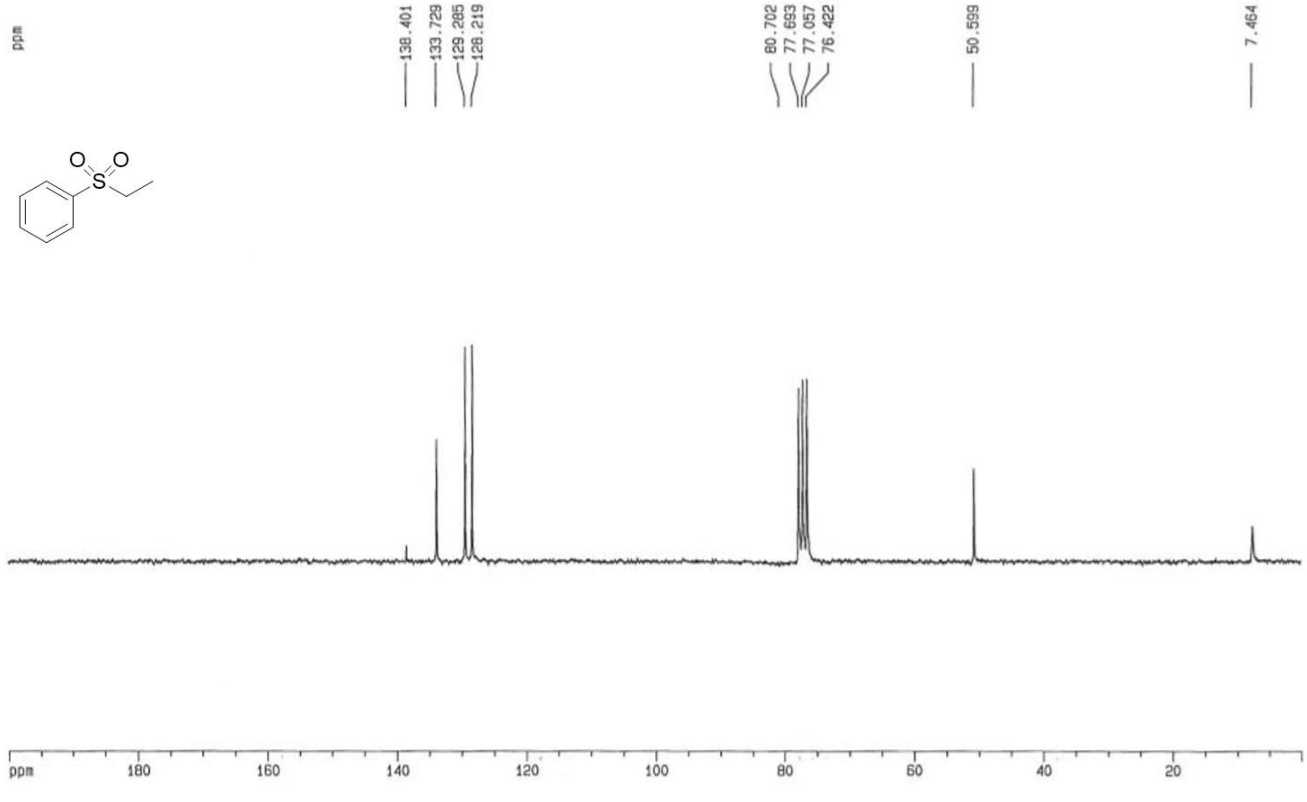
ppm
138.401
133.729
129.285
128.219
80.702
77.693
77.057
76.422
50.599
7.464
ppm
180
160
140
120
100
80
60
40
20

**2b**

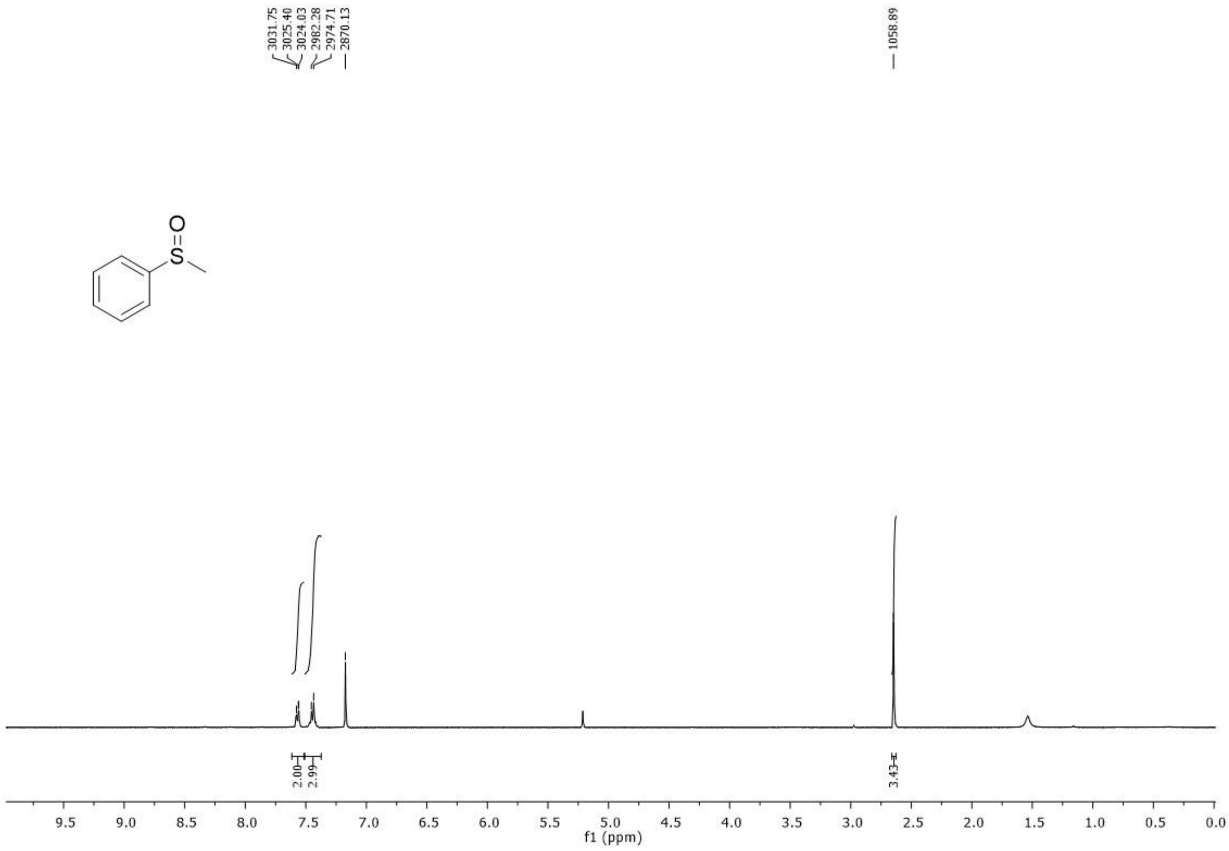
3031.75
3025.40
3024.03
2982.28
2974.71
2870.13
1058.89
2.00
2.99
3.43
9.5
9.0
8.5
8.0
7.5
7.0
6.5
6.0
5.5
5.0
4.5
4.0
3.5
3.0
2.5
2.0
1.5
1.0
0.5
0.0
f1 (ppm)

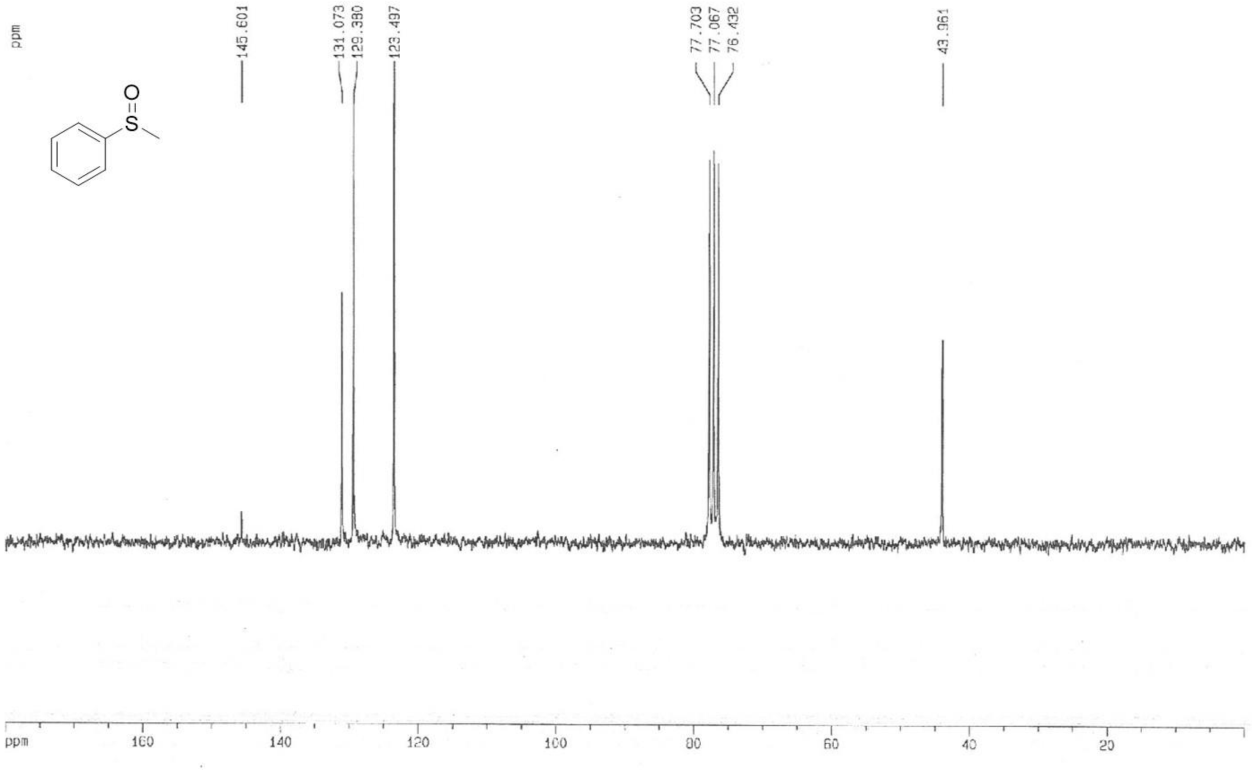
ppm
145.601
131.073
129.380
123.497
77.703
77.067
76.432
43.961
ppm
160
140
120
100
80
60
40
20

3**b**

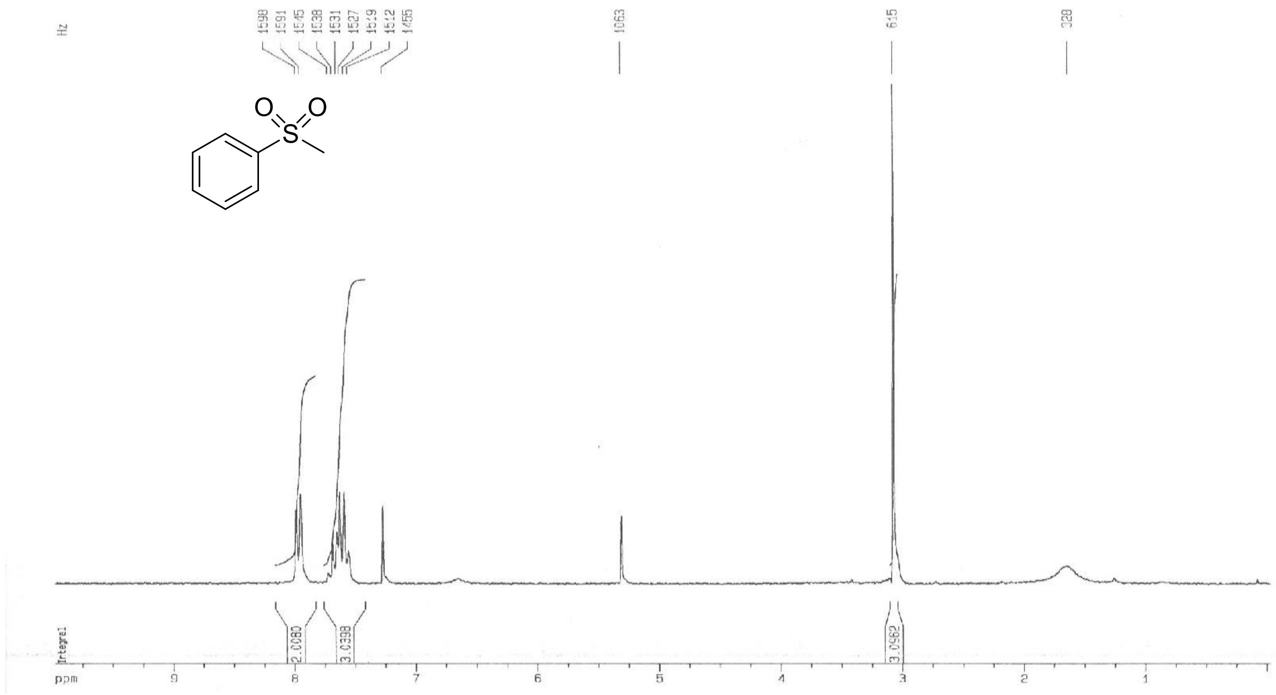
Hz
1598
1591
1545
1538
1531
1527
1519
1512
1455
1063
615
328
Integral
2.0080
3.0398
3.0962
ppm
9
8
7
6
5
4
3
2
1
O
O
S

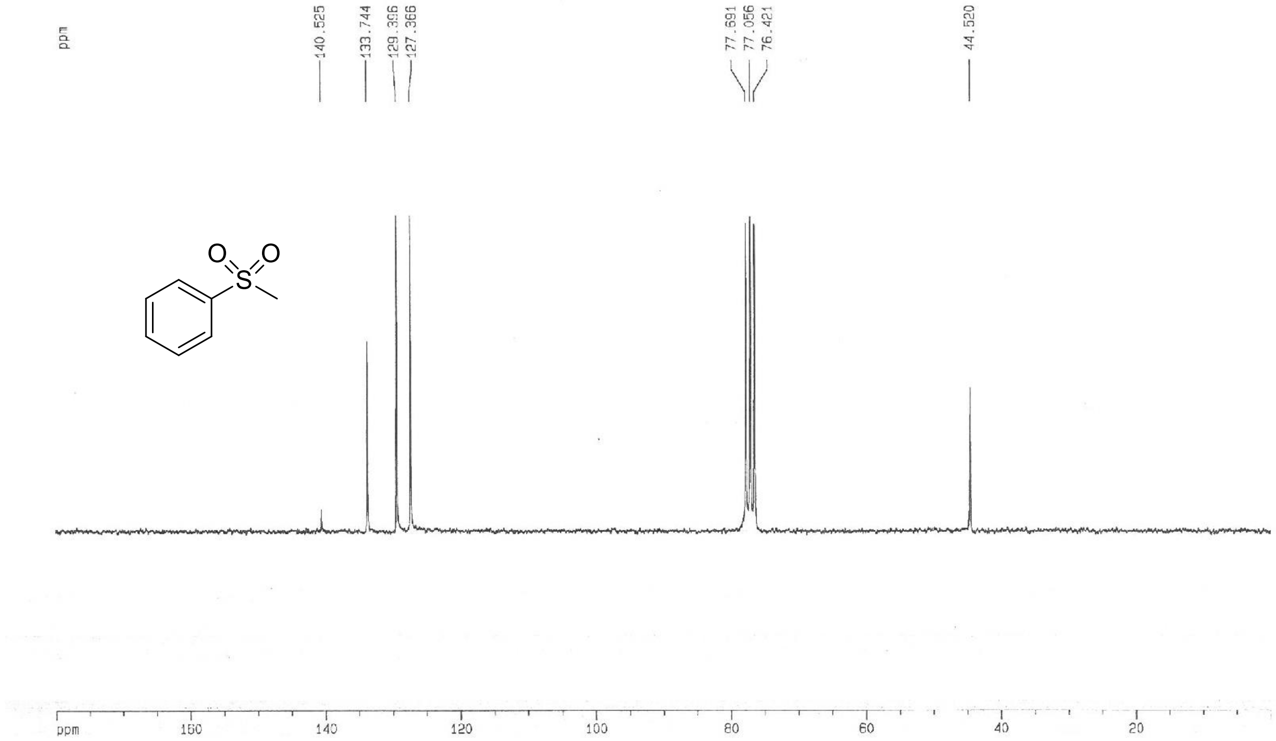
ppm
140.525
133.744
129.396
127.366
77.691
77.056
76.421
44.520
ppm
160
140
120
100
80
60
40
20
O
O
S

**2c**

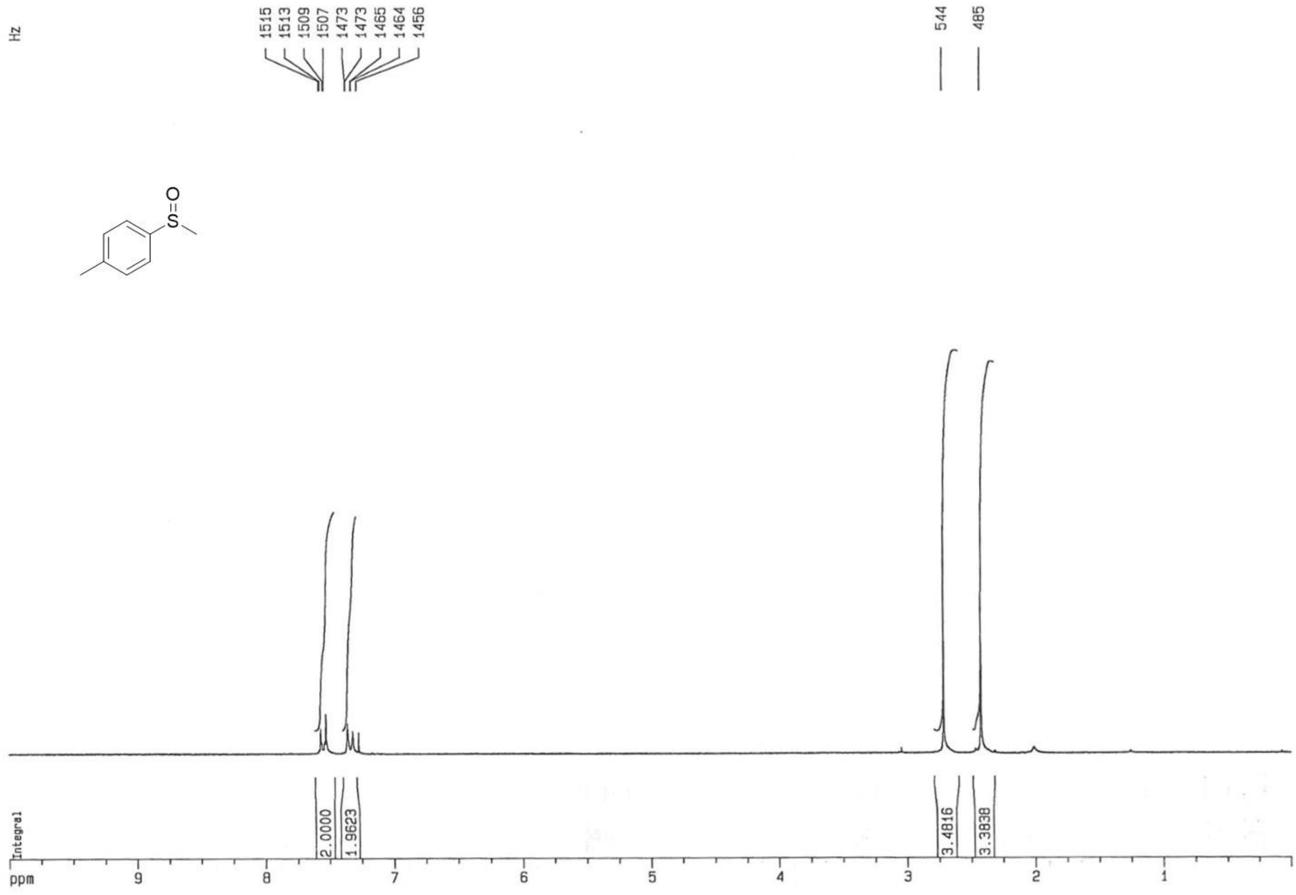
Hz
1515
1513
1509
1507
1473
1473
1465
1464
1456
544
485
Integral
2.0000
1.9623
3.4816
3.3838
ppm
9
8
7
6
5
4
3
2
1

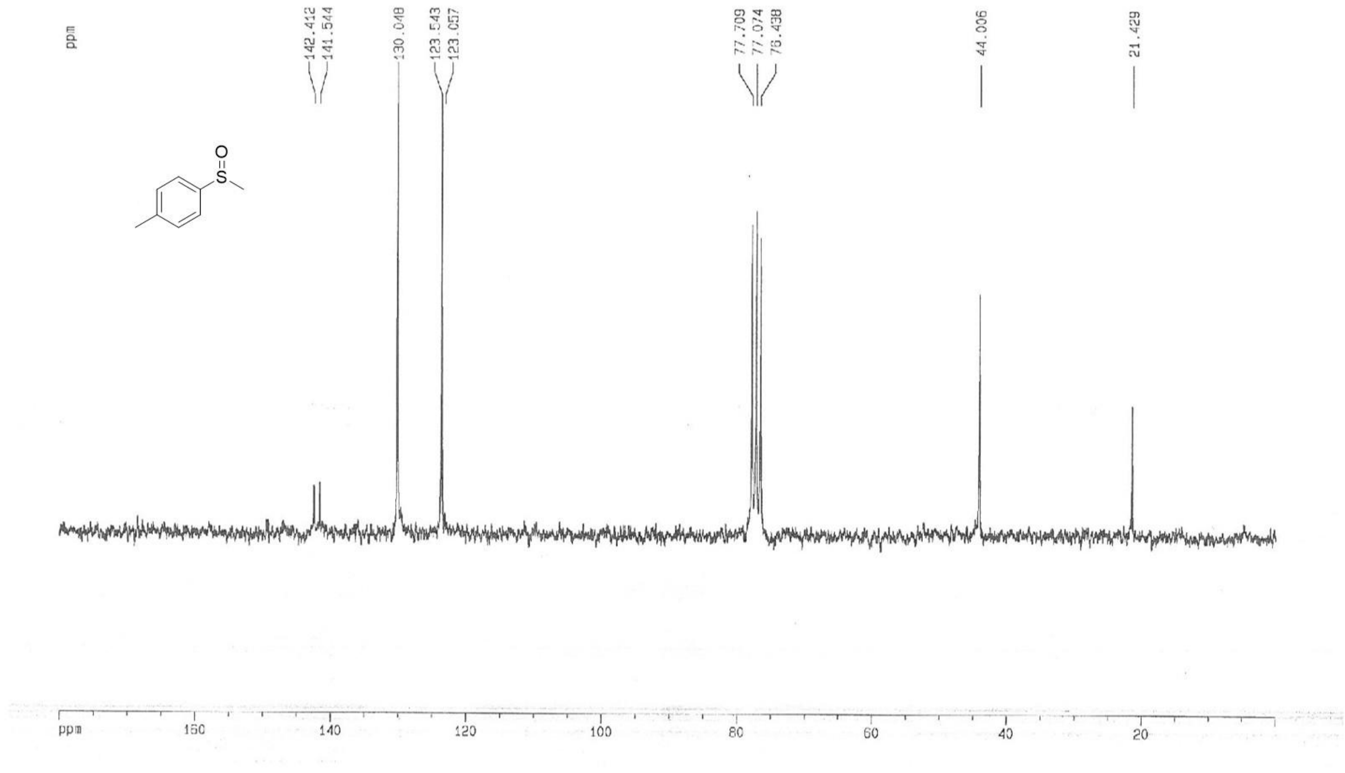
ppm
142.412
141.544
130.048
123.543
123.057
77.709
77.074
76.438
44.006
21.429
160
140
120
100
80
60
40
20

**3c**

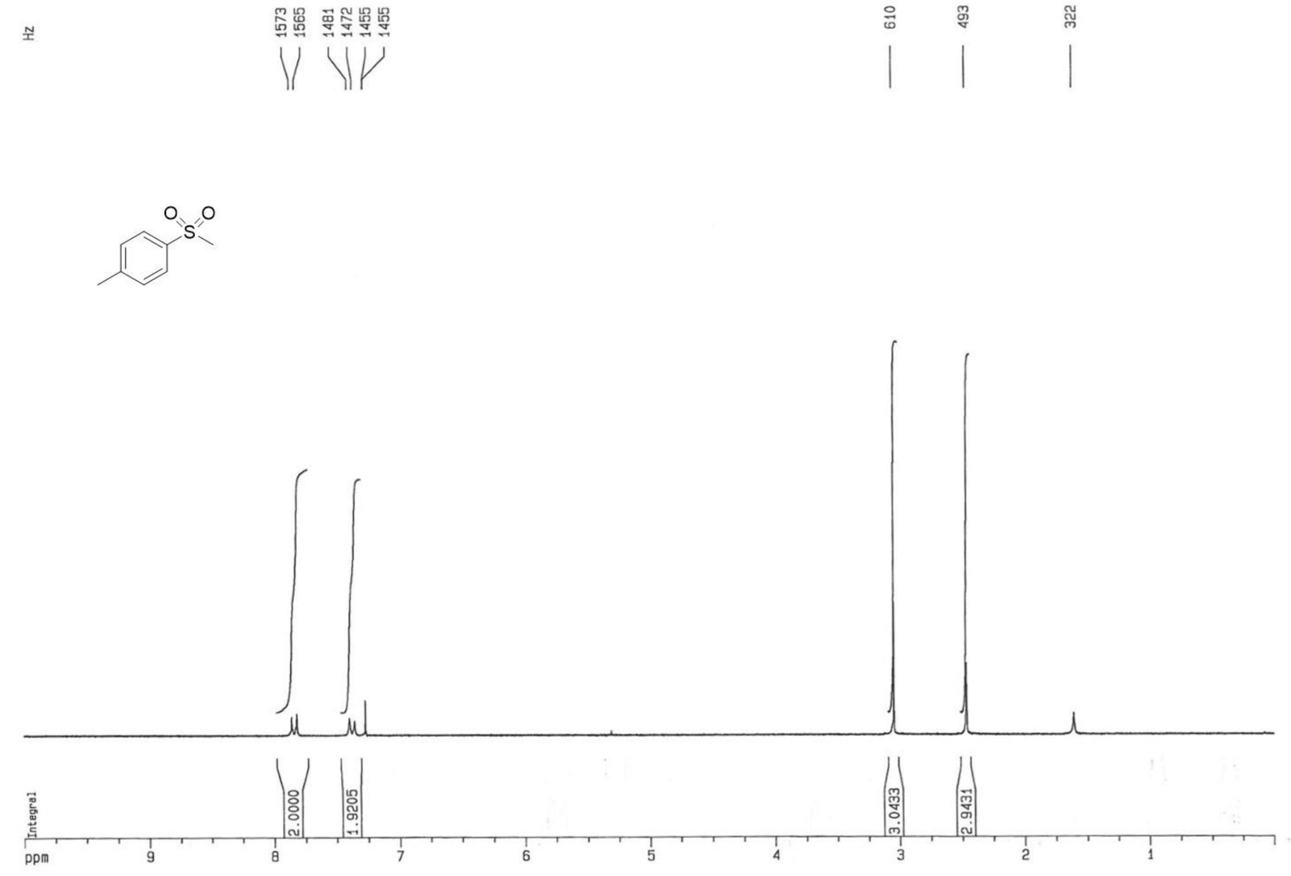
Hz
1573
1565
1481
1472
1455
1455
610
493
322
Integral
2.0000
1.9205
3.0433
2.9431
ppm
9
8
7
6
5
4
3
2
1

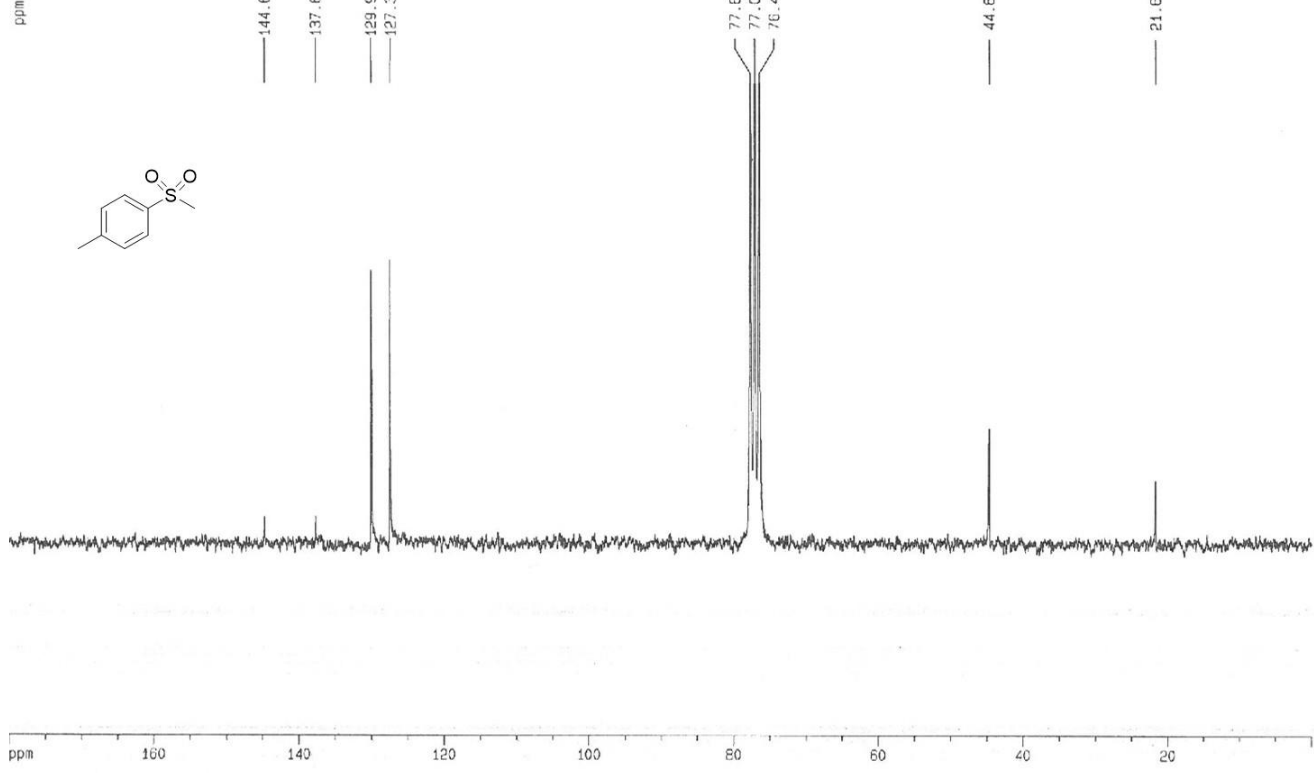
ppm
144.698
137.667
129.970
127.396
77.670
77.035
76.400
44.648
21.662
ppm
160
140
120
100
80
60
40
20

**2d**

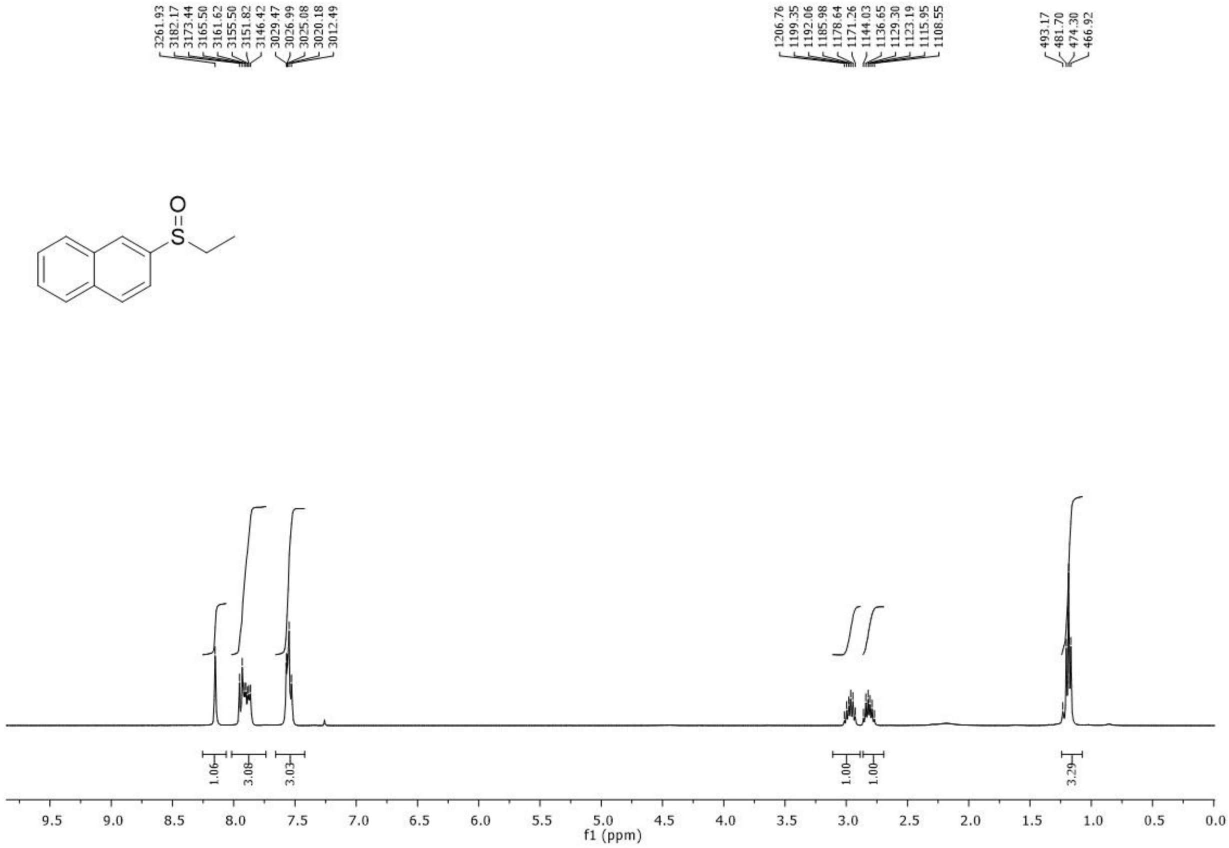
3261.93
3182.17
3173.44
3165.50
3161.62
3155.50
3151.82
3146.42
3029.47
3026.99
3025.08
3020.18
3012.49
1206.76
1199.35
1192.06
1185.98
1178.64
1171.26
1144.03
1136.65
1129.30
1123.19
1115.95
1108.55
493.17
481.70
474.30
466.92
O
S
1.06
3.08
3.03
1.00
1.00
3.29
f1 (ppm)

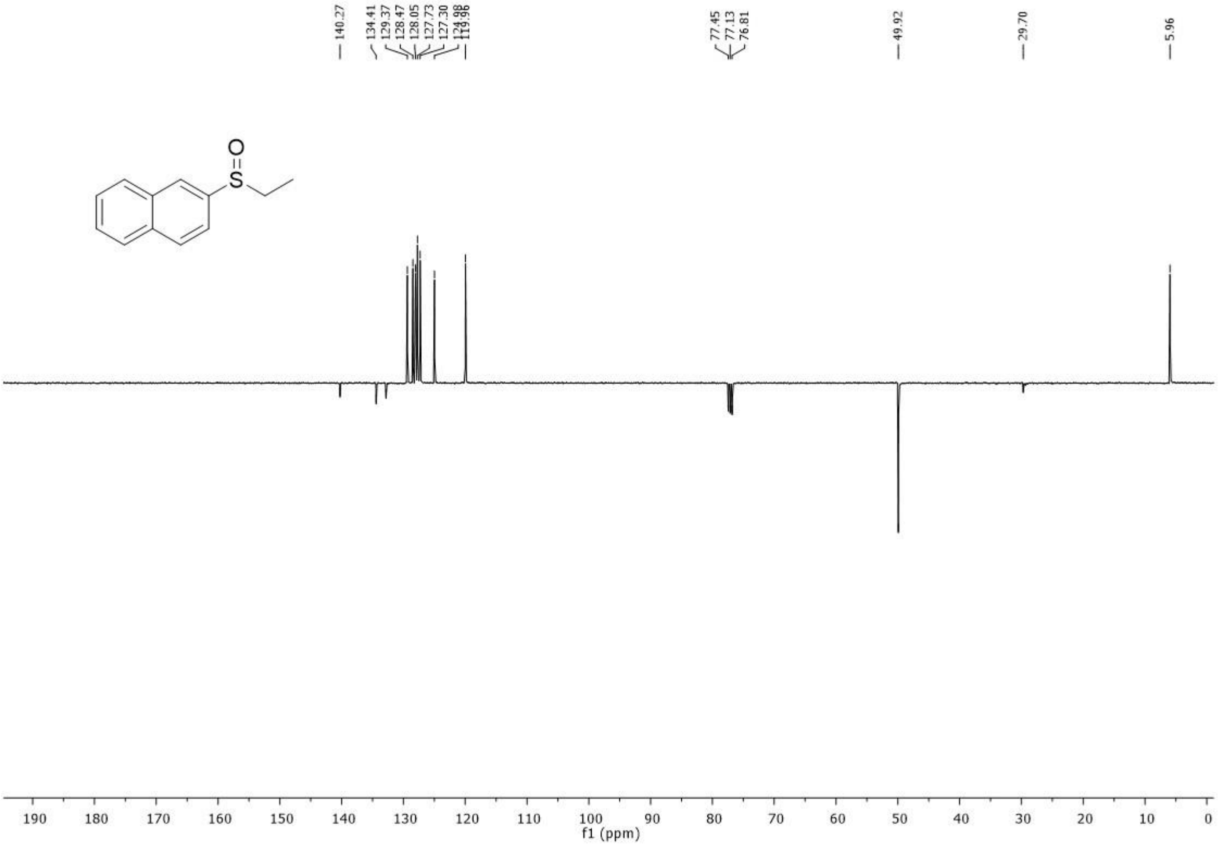
140.27
134.41
129.37
128.47
128.05
127.73
127.30
124.98
119.96
77.45
77.13
76.81
49.92
29.70
5.96
O
S
f1 (ppm)

**3d**

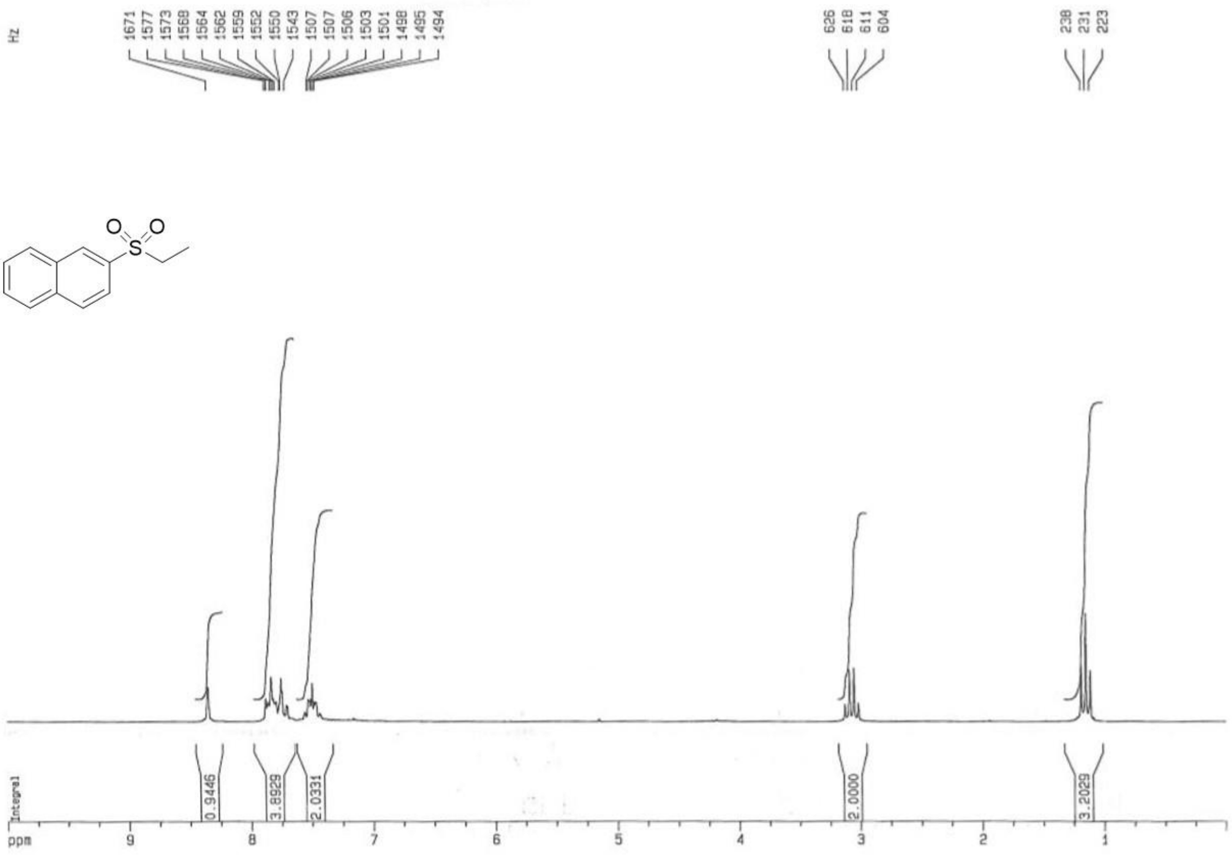

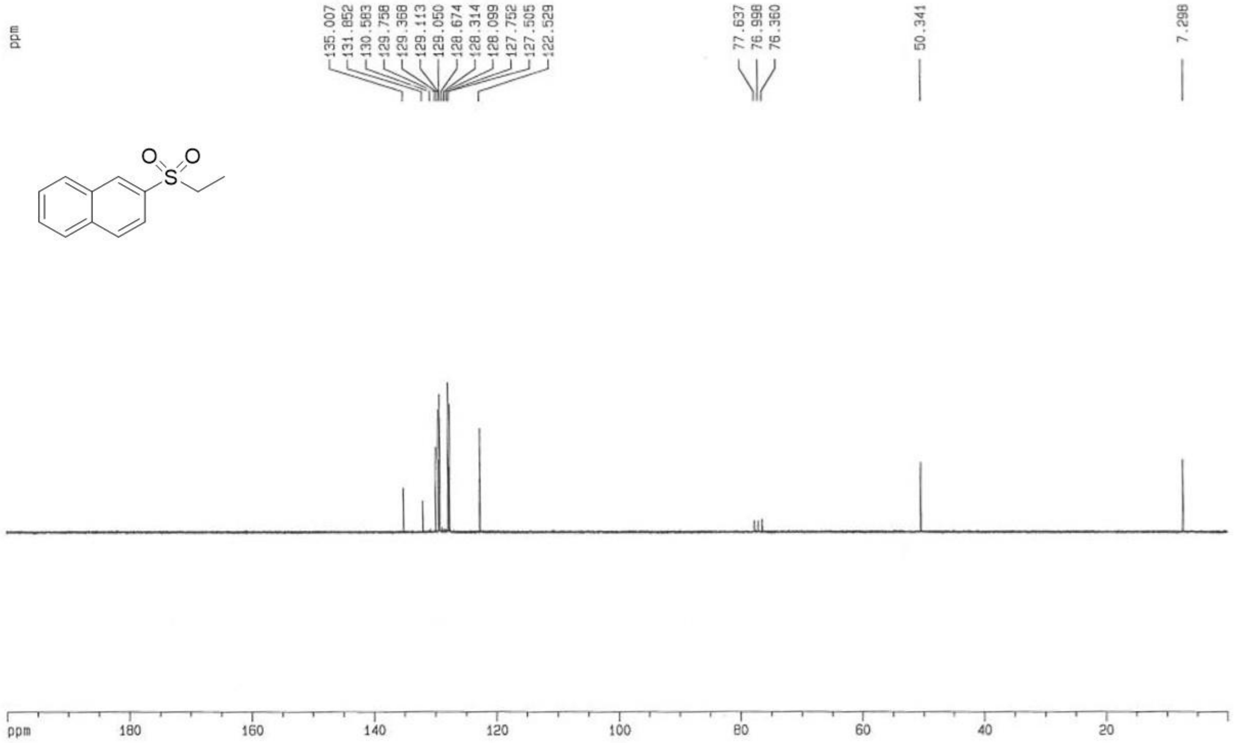

**2e**

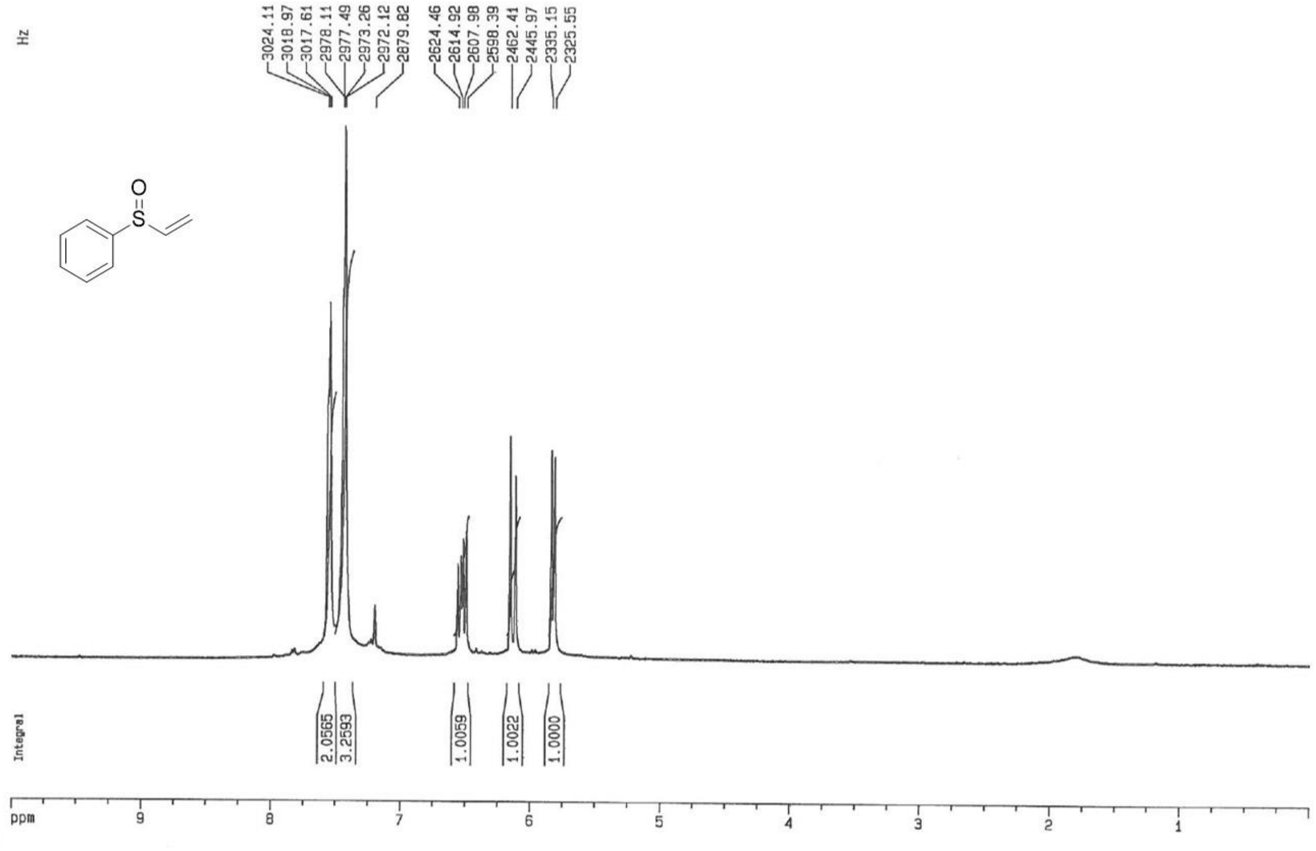
Hz
3024.11
3018.97
3017.61
2978.11
2977.49
2973.26
2972.12
2879.82
2624.46
2614.92
2607.98
2598.39
2462.41
2445.97
2335.15
2325.55
Integral
2.0565
3.2593
1.0059
1.0022
1.0000
ppm
9
8
7
6
5
4
3
2
1

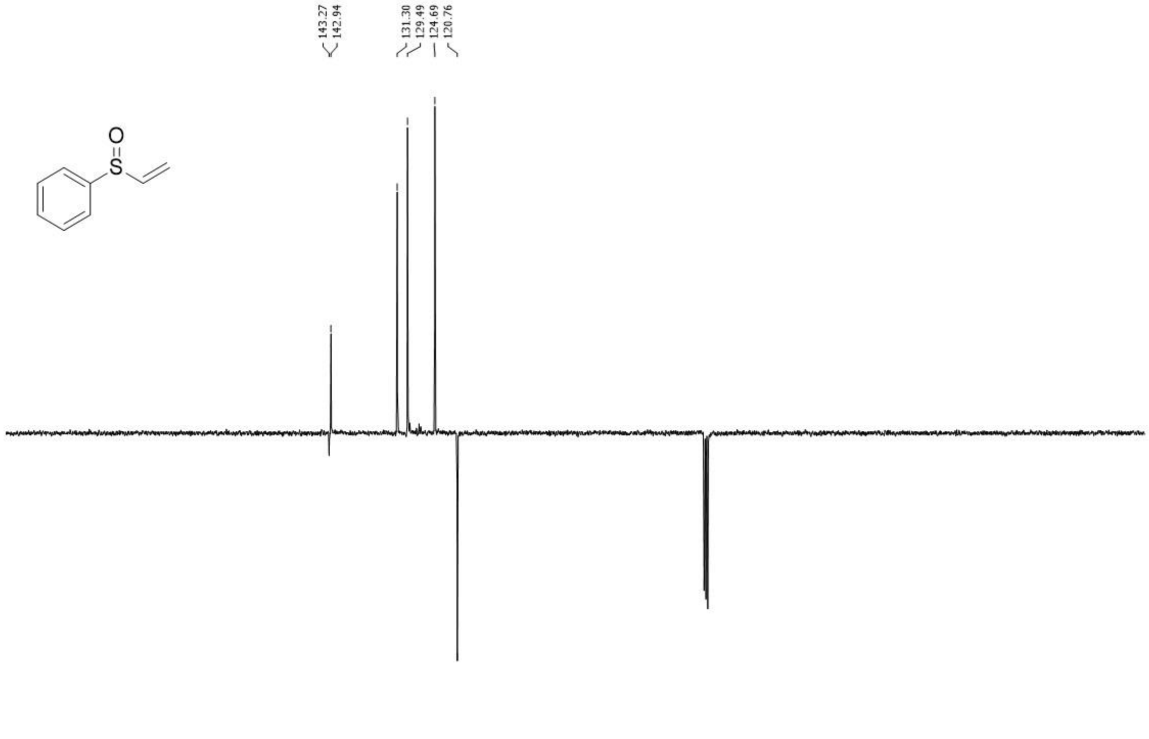
143.27
142.94
131.30
129.49
124.69
120.76
200
190
180
170
160
150
140
130
120
110
100
90
80
70
60
50
40
30
20
10
f1 (ppm)

**3e**

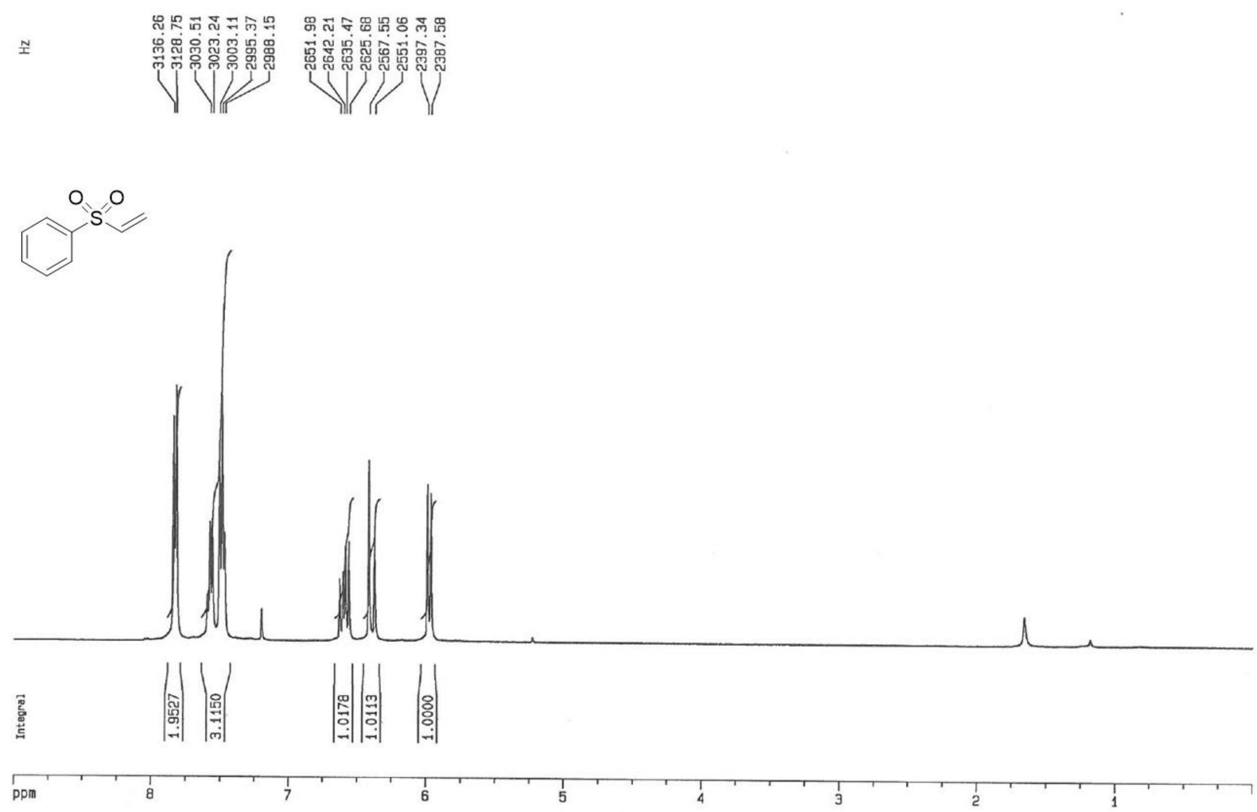
Hz
3136.26
3128.75
3030.51
3023.24
3003.11
2995.37
2988.15
2651.98
2642.21
2635.47
2625.68
2567.55
2551.06
2397.34
2387.58
Integral
1.9527
3.1150
1.0178
1.0113
1.0000
ppm

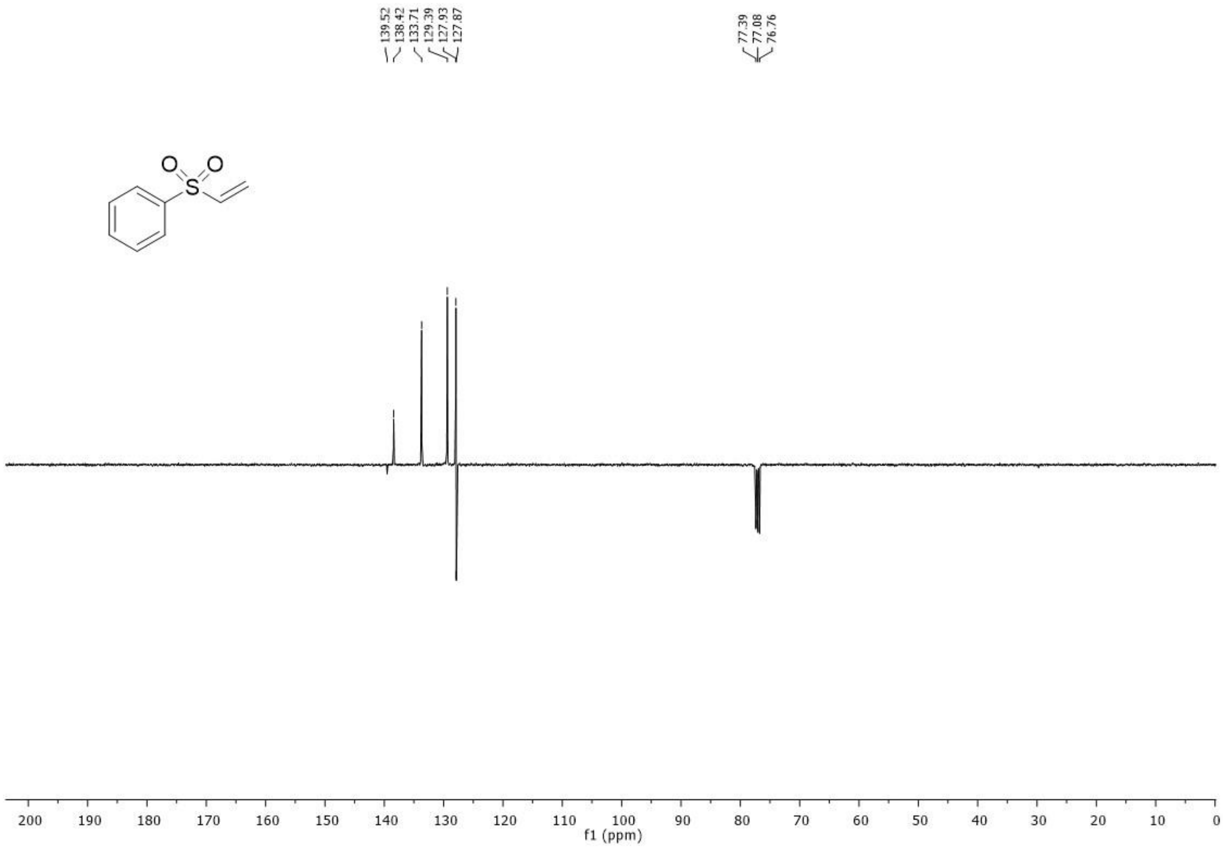
139.52
138.42
133.71
129.39
127.93
127.87
77.39
77.08
76.76
f1 (ppm)

2f

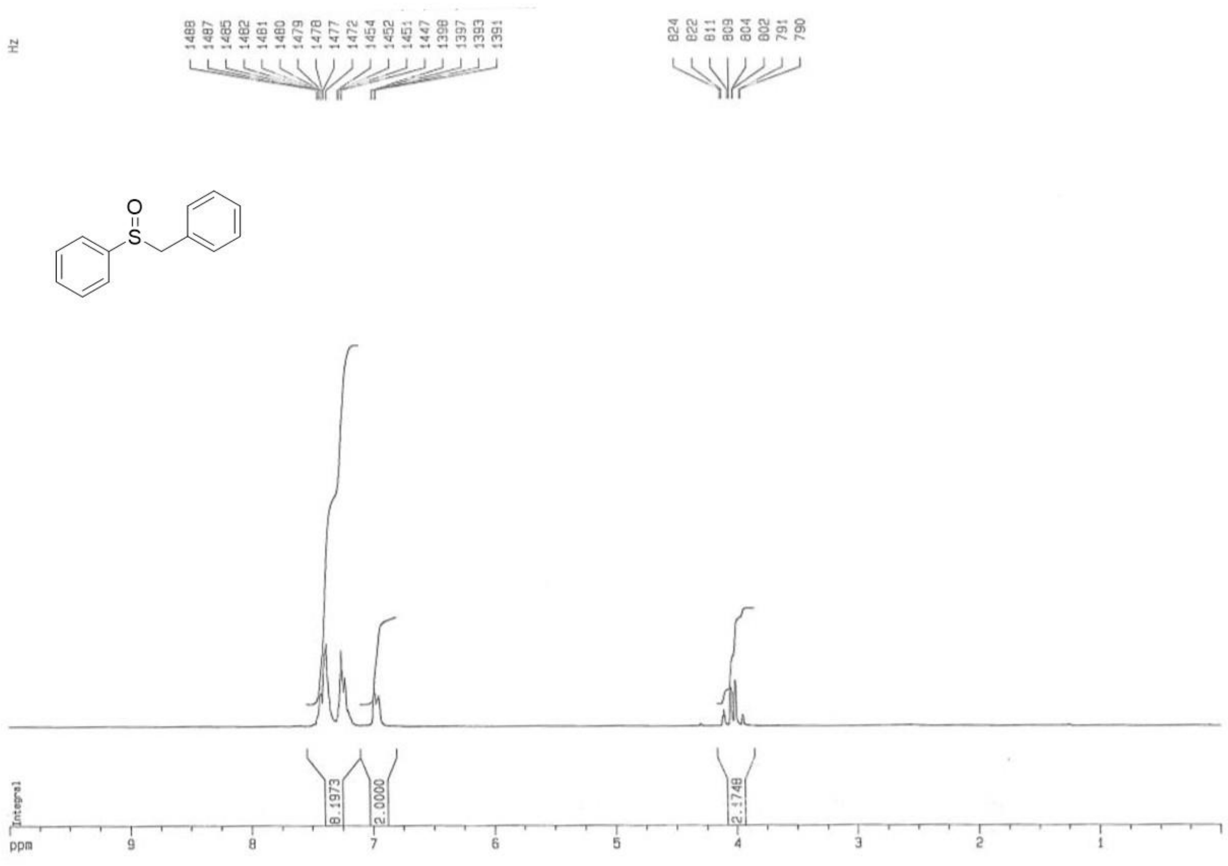
Hz
1488 1487 1485 1482 1481 1480 1479 1478 1477 1472 1454 1452 1451 1447 1398 1397 1393 1391
824 822 811 809 804 802 791 790
O
S
8.1973
2.0000
2.1748
Integral
ppm
9
8
7
6
5
4
3
2
1

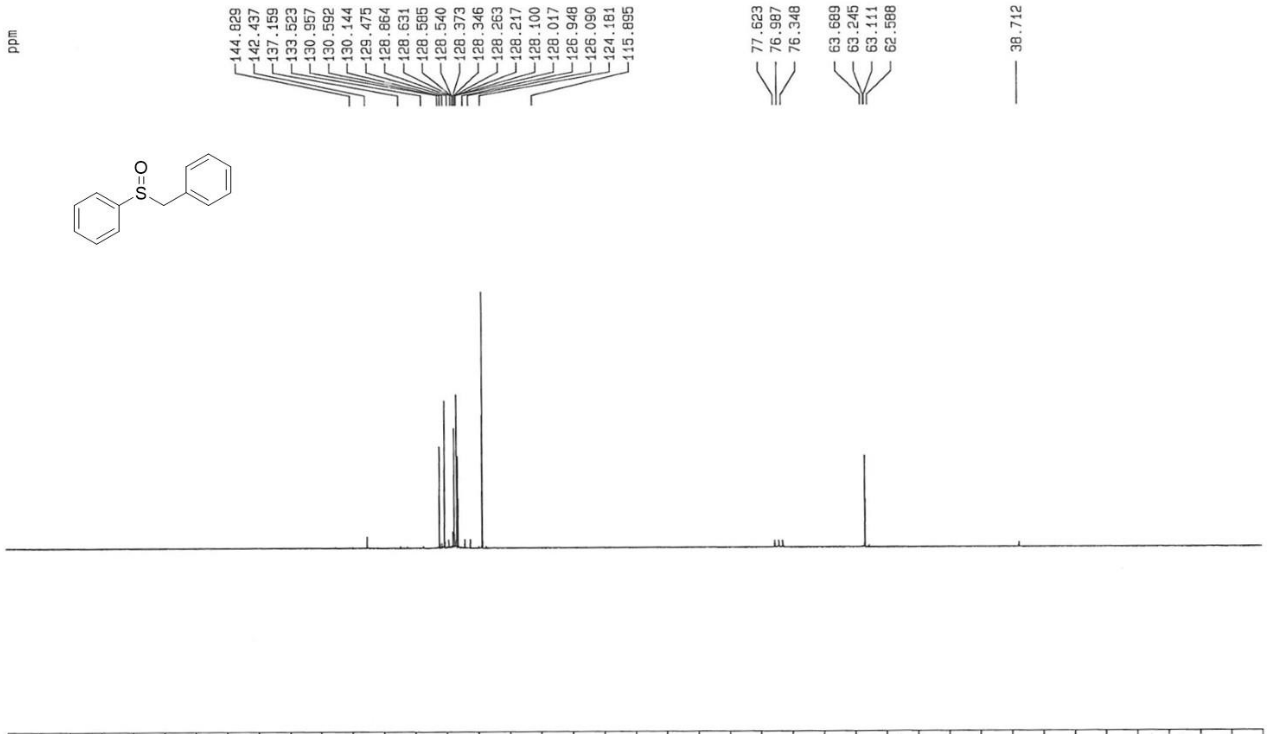
ppm
144.829 142.437 137.159 133.523 130.957 130.592 130.144 129.475 128.864 128.631 128.585 128.540 128.373 128.346 128.263 128.217 128.100 128.017 126.948 126.090 124.181 115.895
77.623 76.987 76.348
63.689 63.245 63.111 62.588
38.712
O
S
ppm
180
160
140
120
100
80
60
40
20

**3f**

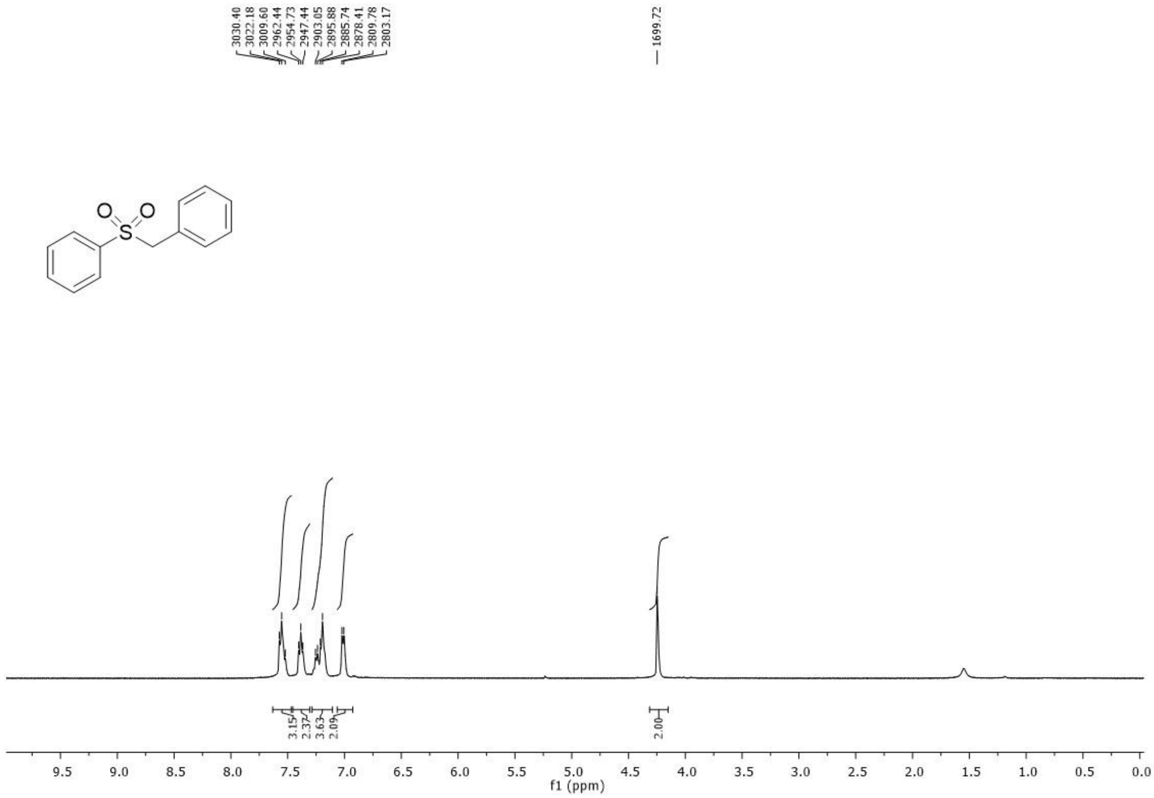

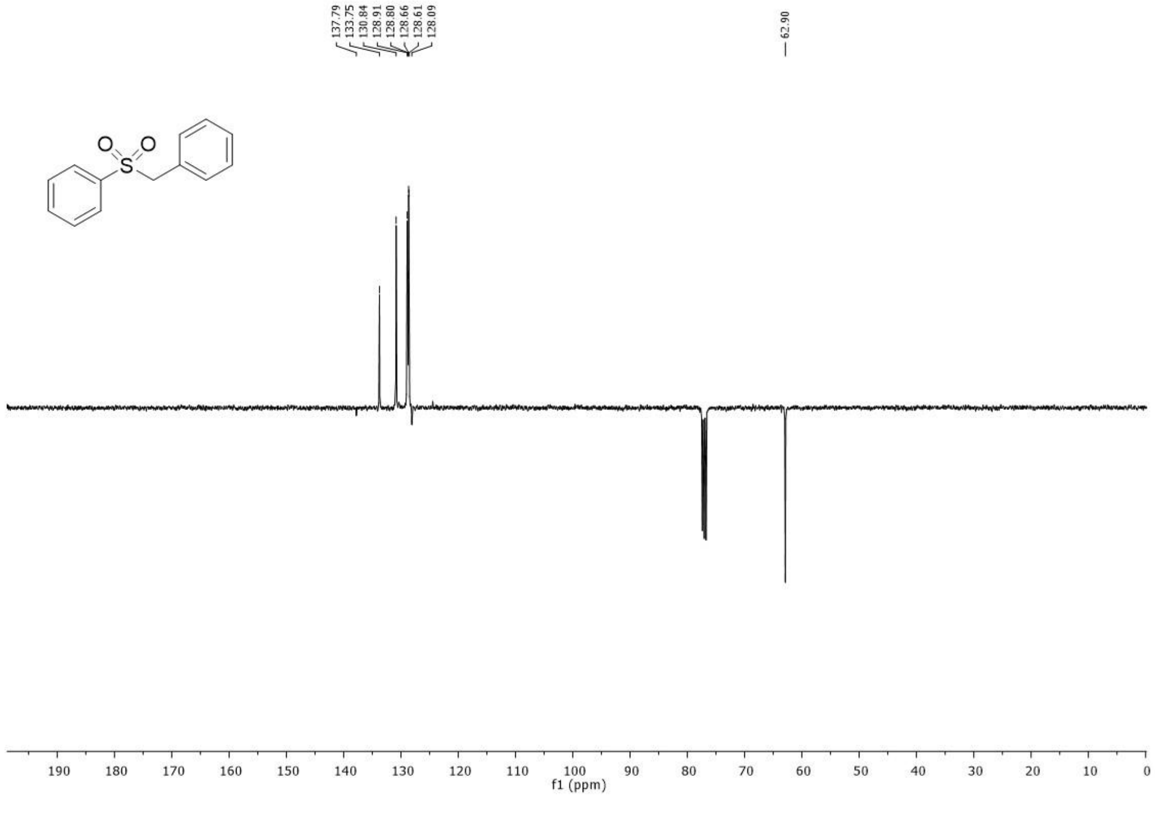

**3g**

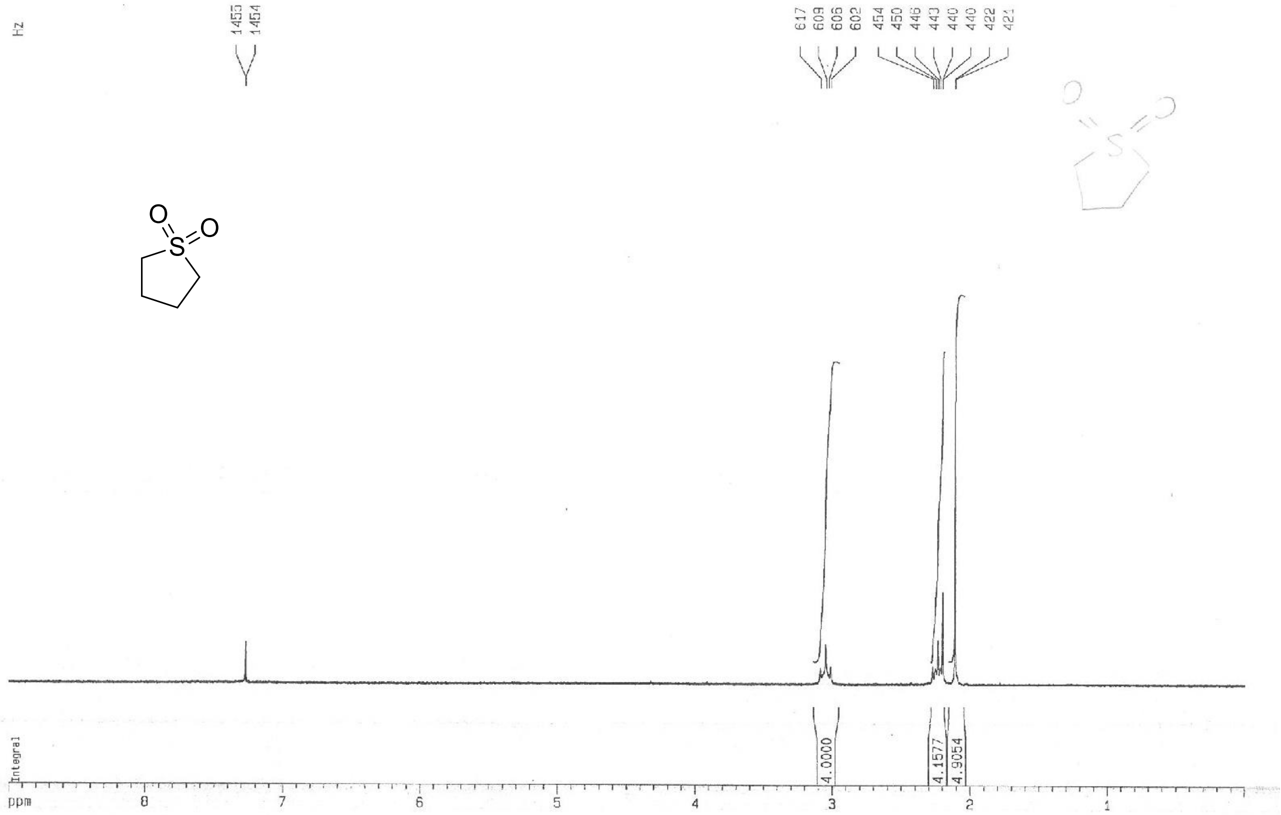
Hz
1455
1454
617
609
606
602
454
450
446
443
440
440
422
421
4.0000
4.1577
4.9054
Integral
ppm
8
7
6
5
4
3
2
1

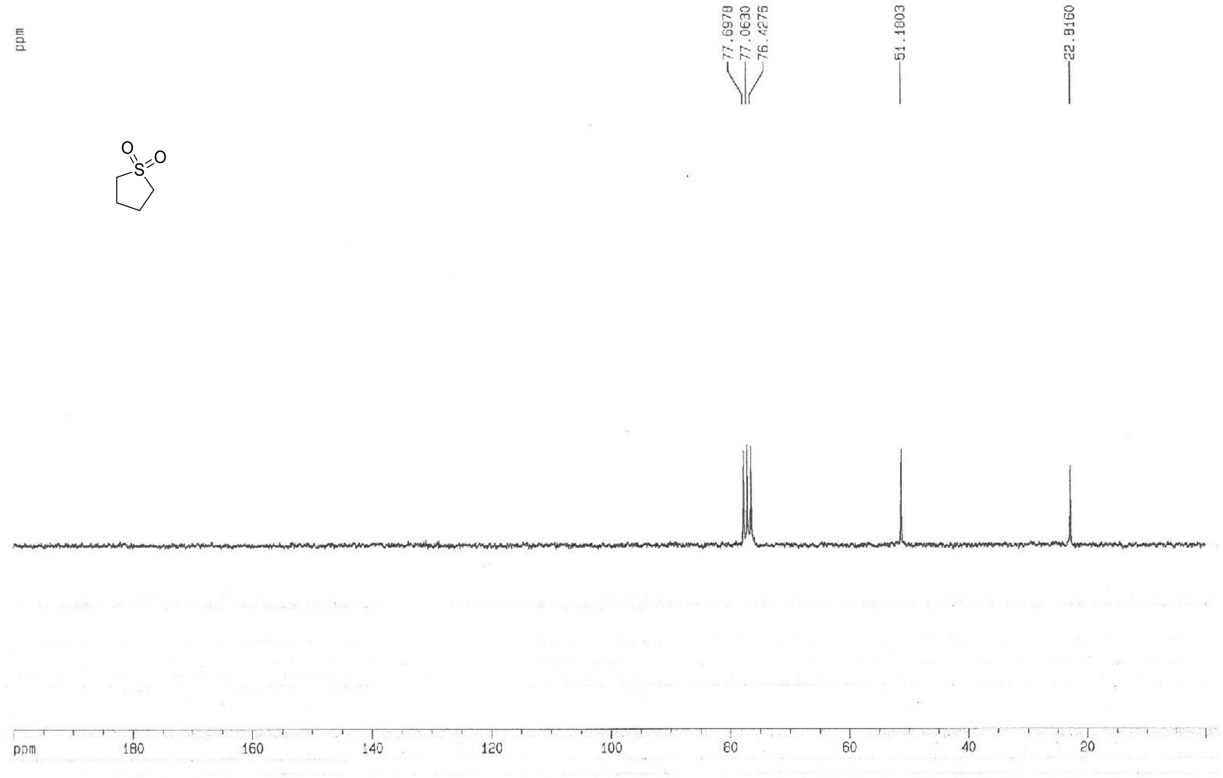
ppm
77.6978
77.0630
76.4276
51.1803
22.8160
ppm
180
160
140
120
100
80
60
40
20

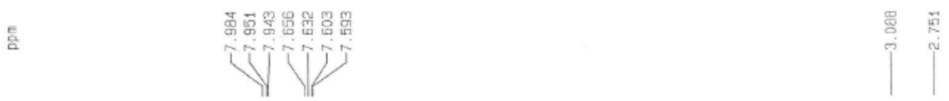


**1H-NMR of the crude product obtained from the scale-up experiment in the oxidation of 1**

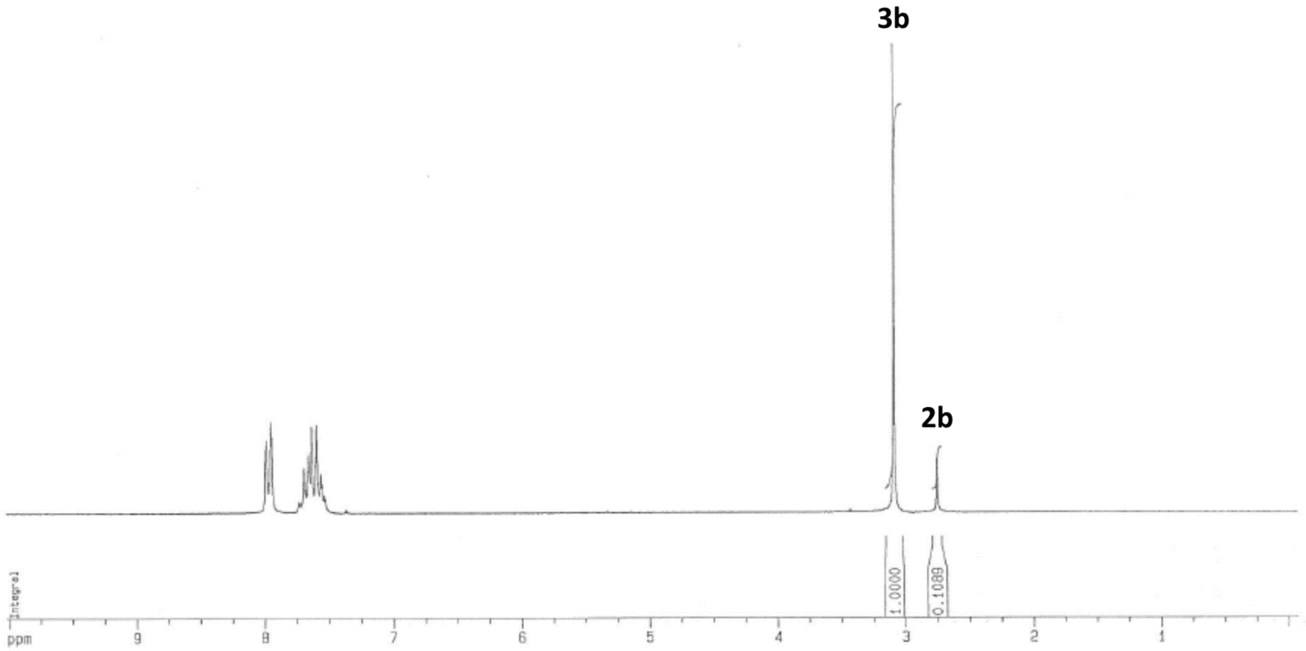

Supplement: Supplementary file 1 [file molecules-25-02711-s001.pdf]
